# Amateur and Recreational Athletes’ Motivation to Exercise, Stress, and Coping During the Corona Crisis

**DOI:** 10.3389/fpsyg.2020.611658

**Published:** 2021-01-27

**Authors:** Franziska Lautenbach, Sascha Leisterer, Nadja Walter, Lara Kronenberg, Theresa Manges, Oliver Leis, Vincent Pelikan, Sabrina Gebhardt, Anne-Marie Elbe

**Affiliations:** Institute for Sport Psychology and Sport Pedagogy, Faculty of Sport Science, Leipzig University, Leipzig, Germany

**Keywords:** corona, sport, athletes, coping, control balance theory, future, support

## Abstract

The COVID-19 pandemic has negatively impacted mobility worldwide. As a corollary, the health of top- and lower-level athletes alike is profoundly reliant on movement and exercise. Thus, the aim of this study is to understand impacts of the COVID-19 pandemic lockdown on athletes’ motivation to exercise and train. In detail, we aim to better understand who (i.e., demographic, sport-specific, and psychological state and trait variables) reported a change in motivation to train due to the lockdown, why they reported lower motivation (i.e., open-ended questions on problems), what they did to help themselves, what support they received from others, and what they are looking forward to after the lockdown (i.e., open questions). Questionnaire data and answers to these open-ended questions were assessed via an online questionnaire, completed by 95 amateur and recreational athletes during the first COVID-19 lockdown in Germany (April to mid-May 2020). Results show that greater numbers of female athletes are less motivated to train in comparison to male athletes (*p* = 0.029). No differences in motivation were found regarding type of sport (individual vs. team sport) and number of competitions during the year. Also, more motivated to train amateur and recreational athletes showed lower athletic identity than athletes who reported no change in motivation to exercise during the lockdown (*p* = 0.03). Additionally, differences in state emotional, perceived stress, and personality variables (i.e., orientation to happiness, volition) were found between athletes who stated that they were less motivated to train compared to athletes who reported no changes in motivation. In particular, closure of sports facilities and social distancing measures were perceived to be highly problematic. Even though athletes received emotional support, organized themselves via routines and schedules, and trained using online tools, they predominately stated that they wished that their coaches would have supported them more. Understanding the impacts of a pandemic-related lockdown on athletes’ motivation, athletes’ coping strategies, and their desired support will help better support them in future crises.

## Introduction

During the first COVID-19 pandemic lockdown, it was as though all movement had come to a standstill. Even though Germany was not impacted as hard as its European neighbors, a nationwide lockdown was announced on March 18, 2020. Schools, restaurants, and sporting facilities were closed and social contacts were regulated at work and prohibited between households. These measures profoundly changed our interactions, including those of the sporting world. Since then, the physical and psychological consequences of COVID-19 pandemic lockdown on athletes have been described in several position papers and editorial statements (e.g., Association for Applied Sport Psychology; International Society of Sport Psychology: Henriksen et al., 2020). Specifically, sport science professionals and scientists have been asked to support athletes in overcoming this *crisis of uncertainty* (Leisterer et al., under review) and to provide guidance both during (e.g., Andreato et al., 2020) and after the crisis. They have, for example, been asked to help prevent health problems due to a potentially tighter competition schedule (Rea, 2020).

To provide adequate guidance from a sport perspective, we first need to adapt theories and models to the context of a lockdown (e.g., see the adapted Scheme of Change for Sport Psychology Practice by Samuel et al., 2020). Further, we need data on psychological variables of (amateur and recreational) athletes relevant to different stages of the COVID-19 pandemic (see Samuel et al., 2020) to gain insights into the mental states of athletes. In detail, this study aims to expand our knowledge on stress, anxiety, and coping during the lockdown (di Fronso et al., 2020, p. 10) and to investigate individuals’ motivation or tendency to become physically (more) active when given the chance (Brand et al., 2020; González-Valero et al., 2020; Maher et al., 2020) during this very unusual time. Current developments underline the increasing relevance of being prepared for such crises and the need for developing appropriate intervention strategies (i.e., second lockdowns, canceled competitions).

### Stress

The stage-A lockdown following the global COVID-19 pandemic has been implicated in athletes’ instability and confusion, strong emotional responses and cognitive appraisal (Samuel et al., 2020). Moreover, the lockdown can be considered a career/transition barrier within a crisis transition (see athletic career transition model in Stambulova, 2011; adapted to corona crisis Stambulova et al., 2020, p. 2). Position papers and editorials published so far emphasize the negative impact of a lockdown on physical and psychological variables for athletes and for the sporting world as a whole (e.g., Andreato et al., 2020; Mehrsafar et al., 2020; see overview by Lim and Pranata, 2020). Stress, anxiety, and/or depression appear to be the most frequently mentioned negative consequences (e.g., Andreato et al., 2020; Frank et al., 2020; Mehrsafar et al., 2020; Schinke et al., 2020; Tingaz, 2020) or as Spitzer summarized it: “When we talk about the psychology of the corona pandemic, we talk about anxiety and loneliness” (2020, p. 279). Additionally, other emotions such as anger, frustration, denial, sadness, helplessness, fear, and/or an enormous sense of loss (AASP (Association for Applied Sport Psychology) Blog, 2020) may be related to the corona crisis. Specifically, interrupted training routines, financial problems (e.g., Andreato et al., 2020), social isolation, and distancing have increased (Schinke et al., 2020). These developments may also include lack of communication among athletes and coaches (e.g., Jukic et al., 2020), reduced training quality and quantity in elite sportswomen (Bowes et al., 2020) and handball players (Mon-López et al., 2020) and the general population (Maugeri et al., 2020). Furthermore, adverse behaviors such as drinking and smoking as well as eating and sleeping problems (e.g., Andreato et al., 2020; Maher et al., 2020), or increased gambling problems also in athletes (Håkansson et al., 2020) are evident. On the other hand, the corona crisis can be considered an *opportunity-provider* according to the corona crisis adapted athletic career transition model Stambulova et al., 2020, p. 6). That is, increased resourcefulness might be beneficial for athletes to overcome obstacles in the long-term. In the same vein, most position statements underline possibilities for personal growth and for stronger relationships that the crisis might offer (e.g., AASP (Association for Applied Sport Psychology) Blog, 2020; Schinke et al., 2020; Wylleman, 2020). These effects have already been empirically shown for an improved coach–athlete relationship (Li et al., 2020).

So far, only a few studies have actually investigated athletes’ mental health in response to the lockdown and the few available results are inconsistent. In detail, it has been shown on a descriptive level that the mental state of 199 sport students from Bulgaria and Russia was not negatively impacted by the pandemic and related social isolation (data assessed in the last 2 weeks of April 2020 in Iancheva et al., 2020). On the other hand, elite athletes are reportedly worried about the future of their sport (66% of *N* = 327) and have reported feeling psychologically worse during the pandemic, according to the measures depression and anxiety (Håkansson et al., 2020). To our knowledge these are the only two studies that have compared data on stress variables prior to and during the COVID-19 lockdown. Results indicate that during the lockdown, perceived stress has increased significantly, and functional and dysfunctional psychobiosocial states have decreased significantly in over 1000 Italian athletes (data assessed April 2020 in di Fronso et al., 2020). On the contrary, however, a study investigating martial arts athletes and other athletes has shown a decrease in perceived stress during compared to the pre-lockdown period (data assessed May-July 2020 in Makarowski et al., 2020). Authors argue that this might be due to habituation, reduced training or competition schedules or a shift in personal priorities (Makarowski et al., 2020). Interestingly, they also found differences between countries. For example, martial arts athletes’ intrapsychic stress was the highest in Lithuania, and significantly higher than in Poland or Romania. Differences in stress perception between male and female athletes were also investigated, and most studies have shown that females report higher levels of perceived stress during lockdown (e.g., di Fronso et al., 2020). In a similar vein, female Olympic and Paralympic athletes have shown higher levels of inflexibility and negative feelings compared to their male counterparts during lockdown (Clemente-Suárez et al., 2020). In a qualitative study, 40% of 18 elite athletes stated that they feel anxious and socially isolated during the COVID-19 pandemic (Tingaz, 2020) whereas 26% of the athletes reported an increase in self-awareness. In detail, they discovered something new about themselves that they were unaware of before (Tingaz, 2020). Overall, it can be stated that the COVID-19 pandemic can be considered both a barrier and a stressor (Tingaz, 2020) to which athletes respond and cope with individually.

### Coping

Coping is defined as how we manage or regulate our emotions (i.e., problem-focused vs. emotion-focused; Lazarus, 2000). How athletes can cope and what measures they should undertake in the face of the crisis have been described in several psychological guidelines (e.g., Batey and Parry, 2020) and in the recommendations of task forces comprised of sport psychological experts, for example in the United States, Canada, and the United Kingdom (for more details see AASP (Association for Applied Sport Psychology) Blog, 2020). Aspects such as acceptance and adaptation, the importance of how to search for information, or monitoring thoughts and feelings have been mentioned as relevant (i.e., top 3 in Bertollo et al., 2020). The use of technological support and favorable living conditions, including healthy eating, having cardio equipment at home, engaging in alternative sport skills training and organized training at home have also been described as useful (e.g., Jukic et al., 2020). Sport psychologists have reported a higher demand for online psychological counseling (Mehrsafar et al., 2020). Techniques that sport psychologists commonly apply are mindfulness, guided performance imagery, re-setting goals, revisiting competition plans, affirmation exercises, and discussions of holistic life balance and values, depending on an athlete’s individual needs (Schinke et al., 2020). In addition, further workshops are currently being developed and evaluated (Leisterer et al., under review).

Again, very few studies have investigated how athletes cope during the crisis and how they can be supported (see also Yang et al., 2020). In the sample of martial arts athletes, a variety of coping strategies emerged: denial, focus on and venting of emotions, alcohol/drug abuse and acceptance (Makarowski et al., 2020). Sport students from Bulgaria and Russia engaged in active planning/cognitive restructuring (i.e., problem-based coping) and emotional calming (i.e., emotion-oriented coping) whereas mentally withdrawing (i.e., distraction-oriented coping) from the situation showed lowest numbers (Iancheva et al., 2020). A similar order of coping strategies can be seen in a sample of 18 elite athletes from Turkey (Tingaz, 2020). Although possibilities were limited, all engaged in physical training (Tingaz, 2020), arguably as emotion-oriented coping. Additionally, athletes trained mentally (33%; i.e., problem-based coping) by watching previous competitions and taking up a new hobby (17%; i.e., distraction-oriented coping). Overall, data on athletes’ coping strategies as well as their stress and anxiety levels during this bizarre time are limited and more detailed studies on the topic are needed (di Fronso et al., 2020, p. 10).

### Motivation to Exercise

“Motivation refers to the extent to which our behavior is selected, directed, energized, and maintained to satisfy a particular motive” (Kazén and Quirin, 2018, p. 15). Motivation, or rather a lack thereof can be understood as the internally driven part of a COVID-19 career barrier (Stambulova et al., 2020, p. 3). In accordance to the definition, this means a lack of movement toward a particular motive (e.g., affiliation, achievement, power; Kazén and Quirin, 2018, p. 15). Scholars are convinced that this barrier will increase in athletes due to the corona crisis. In detail, they refer to loss of motivation (Clemente-Suárez et al., 2020), problems related the topic of motivation (Iancheva et al., 2020), and modifications in motivation (Samuel et al., 2020).

Thus, sport and exercise scientists have particularly emphasized the relevance of physical training and exercise during the pandemic (e.g., Filipas et al., 2020; Mehrsafar et al., 2020; Paoli and Musumeci, 2020). For example, a decline in physiological functions such as musculoskeletal, neuromuscular, respiratory, and the cardiovascular systems as well as a decrease in physical capacities such as power, strength, flexibility, speed, and endurance may result (see Lim and Pranata, 2020, p. 3). Not only does such a decline lead to an increased risk of injury (Lim and Pranata, 2020) but it may eventually have a negative impact on mental health (Pinto et al., 2020; Slimani et al., 2020).

In this vein, research has shown that frequency of exercise is positively related to mood during the lockdown (Brand et al., 2020). Also, physical activity in college students was positively associated with positive affect independent of the stressful life event (i.e., COVID-19 pandemic; Maher et al., 2020) as well as to mental well-being in the general population (Maugeri et al., 2020). In addition, the mental health status during the first COVID-19 lockdown was better in athletes than in non-athletes, providing support to the notion that physical activity may help protect mental health during times of crisis (Şenışık et al., 2020). Finally, retrospectively assessed data show that individuals with reduced physical activity due to the pandemic are the ones that report a significant decline in well-being, in comparison to pre-COVID-19 (Mutz, 2020). This all underlines the importance for (amateur and recreational) athletes to maintain their motivation to train and exercise. Moreover, this supports our study aims to investigate how many athletes maintain motivation and how they differ (i.e., demographic, sport-specific, and psychological state and trait variables) from athletes who fail to maintain their motivation to exercise.

### Personality and Motivation to Exercise

There are several theoretical approaches that (a) provide explanations for a potential change in motivation to exercise (i.e., amotivation according the organismic integration regulation continuum within the self-determination theory, SDT by Deci and Ryan, 2000) and that (b) provide a rational for investigating psychological trait variables in the context of changed motivation, due to the lockdown (i.e., personality systems interaction theory, PSI by Kuhl, 2018; approach and avoidance temperament as personality structure by Elliot and Thrash, 2010).

In reference to SDT, it can be reasoned that due to the lockdown the autonomy to decide for example where, when, and with whom an athlete wants to train dissolves; the relatedness to others for example team mates or the coach vanishes, and finally feelings of competence might be endangered due to absence of a training schedule or the phenomenon of training without a coach. This all may decrease intrinsic motivation–due to the frustration of autonomy, relatedness, competence–and increase the risk of amotivation–due to missing opportunities to perceive competence or value.

According to PSI theory, an excessive increase in stress is characterized by low positive affect and high negative affect, and leads to a decrease in self-regulatory capacity (for further details please see Kuhl, 2018). For example, accessing the extension memory system, including stored action plans (e.g., going to exercise after school/work) as well as the behavioral control which is relevant for executing behavioral routines (e.g., practice went by in no time) needs more self-control or volitional components (Kazén and Quirin, 2018) and thus, not just motivation decreases but eventually, the execution of the behavior (i.e., exercise) will decline.

Finally, approach–avoidance is a motivational distinction (Lewin, 1935) and has been defined as a basic dimension of personality. Individuals with a high approach temperament are high in extraversion, positive emotionality and employ a behavioral activation system (Elliot and Thrash, 2010). During lockdown, these individuals could either experience no change or an increase in motivation to exercise. On the other hand, individuals with avoidance temperament are high in neuroticism, negative emotionality, possess a behavioral inhibition system (Elliot and Thrash, 2010) and might experience a decrease in motivation to train during lockdown. Overall, stress perception might differ between individuals and thus might impact motivation to exercise differently (PIS theory). Also, this impact of stress perception on motivation, might be modulated by differences in personality trait variables such as volitional components (see PSI theory) or extraversion, neuroticism, and anxiety as well as happiness (see approach and avoidance temperament). Thus, in a first step we aim to identify possible differences in these state and trait variables based on grouping individuals depending on their self-reported change in motivation from prior to, to during the COVID-19 lockdown.

So far, research during the COVID-19 crisis has investigated aspects of motivation only indirectly. In detail, it has shown that motivational aspects, which belong to the psychological modalities (i.e., motivation, emotion, cognition, volition) of the biopsychosocial states, have decreased in athletes (see di Fronso et al., 2020). In amateur athletes, rate of commitment to sport has remained high at 61.6% (30.7% moderate; 7.7% low commitment; see Angosto et al., 2020). Another study showed that for the period before to during the confinement, goals shifted and predict the intention to exercise during confinement (Mascret, 2020). In detail, on average, self-approach goals (i.e., improving oneself) decreased and self-avoidance goals (i.e., avoiding regression) increased during the confinement in almost 700 French athletes. Both goals predicted intention to exercise during confinement positively. Thus, the author concludes that situationally dependent adaptation of goals might be beneficial. Data from almost 14,000 individuals from Europe, Asia, as well as South and North America confirm this assumption and show that 44.2% reported no change in their exercise frequency during the COVID-19 lockdown and 31.9% increased their exercise (Brand et al., 2020). However, also 23.7% decreased their exercise frequency. Self-reported data from 631 physically active Germans show that 9% increased their physical activity, 42.4% maintained their level similar to Brand et al. (2020), and 48.7% reduced their level of physical activity (Mutz, 2020). However, postponement of goals such as participating in certain competitions or qualifications might lead to a decrease in motivation to train and exercise (e.g., AASP (Association for Applied Sport Psychology) Blog, 2020; Rea, 2020). Yet, as motivation is controllable, Stambulova and colleagues are convinced that sport psychologists could provide support in such cases (2020, p. 3).

### Athletic Identity

Finally, one trait variable of interest is athletic identity, which has been argued makes athletes a special group that may be especially vulnerable during such a crisis due to their high athletic identity (Batey and Parry, 2020). This is considered to be true for high-performance athletes, amateurs and professionals alike (Schinke et al., 2020). Athletic identity is a part of one’s self-concept, is considered to be relatively stable and is defined as the degree to which a person identifies with the role of being an athlete (Brewer et al., 1993). Due to social isolation, a strong, potentially narrow athletic identity can be critical as it may include increased anxiety and other mental health concerns (Henriksen et al., 2019). With regard to the construct of athletic identity and in line with this assumption, Costa et al. (2020) found a positive relation between athletic identity and rumination as well as catastrophizing in over 1000 Italian athletes during the corona lockdown in late April and early May. However, the question remains whether there is a difference in athletic identity, depending on athletes’ motivation to train and exercise during a lockdown.

### The Present Study

So far, little is known about (amateur and recreational) athletes’ psychological reactions (i.e., motivation, stress, emotion, coping) to the COVID-19 pandemic lockdown. Further, we lack knowledge about the impact of the lockdown on athletes’ motivation to train and exercise. However, lack of exercise can lead to physical and psychological damage (see Mehrsafar et al., 2020). Thus we aim to identify amateur and recreational athletes who have experienced a change in motivation due to the lockdown and who may need targeted support (Stambulova et al., 2020). In detail, we aim to better understand precisely who (i.e., demographic, sport-specific and psychological trait and state variables) has experienced a change in motivation. Further, we aim to explore the problems that amateur and recreational athletes experienced, what they did to help themselves, what support they received from others, which support they missed and what they look forward to following the lockdown (i.e., open questions). Thereby, our combined approach of open and closed questions overall aims to provide detailed information on amateur and recreational athletes’ psychological state.

While we are able to put forward hypotheses for most variables, the remaining variables will be exploratory. In detail, we assume that there will be fewer females motivated to train than males due to higher levels of anxiety reported by females, and the relation of anxiety to motivation (Elliot et al., 2013). Older athletes should be less stressed (based on standard values of the general population, see Klein et al., 2016) and thus, their motivation should be less affected (see PSI theory). Regarding type of sport, we expect no differences between individual and team sport athletes based on findings by di Fronso et al. (2020). Athletes with higher training hours under normal circumstances should react less stressed (see stress-buffer hypothesis, e.g., Gerber et al., 2018) and thus, their motivation should be less affected (see PSI theory). Athletes with more competitions per year should experiences a larger decrease in motivation (see SDT).

Athletic identity will be the lowest in athletes who experience no change in motivation due to less rumination (see Costa et al., 2020). Trait anxiety is assumed to be the lowest in athletes who experience no change in motivation (see approach and avoidance temperament). Trait happiness is expected to be the highest in athletes who experience no change in motivation (see approach and avoidance temperament). Volitional components will be the highest in athletes who experience no change in motivation (PSI theory). Athletes high in neuroticism will experience a decrease in motivation to exercise, whereas athletes high in extraversion will show no change in motivation or an increase (see approach and avoidance temperament). Other personality traits (i.e., openness, conscientiousness, agreeableness) and coping strategies that might play an important role in such a crisis, for example in dealing with risks and its assessment (Funke, 2020) will be explored.

The level of perceived stress and negative emotions during the lockdown will be higher in athletes who experience a change in motivation in comparison to athletes who do not (see Tingaz, 2020; PSI theory). Conversely, positive emotions during the lockdown will be higher in athletes who experience no change in motivation (see PSI theory).

Finally, we will explore open questions regarding problems, coping strategies, wanted and received support and athletes’ joyful anticipations for the time following lockdown. However, based on the athletic career transition model (in Stambulova, 2011) and based on open questions related to the different topics, we expect that both negative and positive aspects will be mentioned by the athletes.

## Materials and Methods

### Participants and Procedure

Prior to the study, no calculations with respect to sample size was performed. Participants were recruited via social media and personal networks, and participated voluntarily without financial compensation. Unipark web-based survey software was used. One hundred eighty-three athletes started filling out the questionnaire and 51.91% finished with a mean processing time of 21 min.

A total of 95 German athletes (51 females, 44 males; *M_*age*_* = 22.03, *SD_*age*_* = 3.63) completed the cross-sectional online questionnaire during the COVID-19 lockdown in Germany (from April to mid-May 2020). Participants performed individual sports (*n* = 45) and team sports (*n* = 43) or both (*n* = 4). In detail, soccer (*n* = 13), volleyball (*n* = 11), fitness (*n* = 9), track and field (*n* = 8), triathlon (*n* = 7), strength training (*n* = 6), dancing (*n* = 5), combat sport (*n* = 5), handball (*n* = 4), CrossFit (*n* = 3), “diverse” (*n* = 3), gymnastics (*n* = 2), badminton (*n* = 2), rowing (*n* = 2), swimming (*n* = 2), cycling (*n* = 2), running (*n* = 2), floorball (*n* = 2), acrobatics (*n* = 2), table tennis (*n* = 1), water polo (*n* = 1), biathlon (*n* = 1), tennis (*n* = 1), climbing (*n* = 1), endurance (*n* = 1), fencing (*n* = 1), ice hockey (*n* = 1), basketball (*n* = 1), beach volleyball (*n* = 1), cross-country skiing (*n* = 1), pole dance (*n* = 1), and canoe slalom (*n* = 1) were mentioned.

Athletes practiced 8 h per week (*SD* = 4.5 h) and had on average 12 competitions per year (*SD* = 13.9) prior to the lockdown. The majority of sampled participants were amateur athletes (i.e., recreational athletes: *n* = 39; athletes competing on a regional level: *n* = 34). Only a few athletes competed on national (*n* = 11) and international (*n* = 11) levels. Almost all participants were students at university level (*n* = 90), two still went to high-school, and three were employed (i.e., one as a teacher, two provided no further information). Please see Tables 1, 2 for details on demographic and sport-specific data.

**TABLE 1:** Demographic and sport-specific data for the complete sample (*N* = 95) dependent on motivational change to exercise (less: *n* = 33; same: *n* = 39; more: *n* = 23).

	**Change in motivation to exercise from**			
	**before to during lockdown**			
	
	**Less (*n*)**	**Same (*n*)**	**More (*n*)**	**Total (*n*)**
**Gender**				
Female	23	15	13	51
Male	10	24	10	44
Total	33	39	23	95
**Type of Sport**				
Individual	15	20	10	45
Team	15	17	11	43
Both	2	2	0	4
Total	32	39	21	92

**TABLE 2:** Demographic and sport-specific data for the complete sample (*N* = 95) dependent on motivational change to exercise (less: *n* = 33; same: *n* = 39; more: *n* = 23).

**Change in motivation to exercise**	***M***	***SD***	***Min***	***Max***
**Age (in years)**				
Less	21.85	3.59	15	34
Same	22.64	4.06	18	39
More	21.26	2.80	17	32
Total	22.03	3.63	15	39
**Training duration (hours/week)**				
Less	8.73	5.29	3	24
Same	9.14	4.41	4	21
More	5.85	2.36	2	11
Total	8.20	4.53	2	24
**Competitions/year**				
Less	11.79	15.99	0	70
Same	15.08	13.66	0	48
More	08.26	9.84	0	30
Total	12.28	13.86	0	70

### Measures and Material

#### Demographic and Sport-Specific Information

Participants were asked to report their age (in years), gender (male vs. female vs. diverse), the type of sport they practice, how often they train under normal circumstances (in hours), and finally, the number of competitions they participate in per year. Please see Tables 1, 2 for details on demographic and sport-specific data.

#### Motivation to Exercise

Motivation to exercise and train was assessed via a single item: “How motivated are you to train in the current situation (COVID-19 pandemic) compared to normal circumstances?” In accordance with Brand et al. (2020), we were more interested in generally perceived changes in exercise motivation. Athletes answered *less*, *same* or *more*.

#### Psychological Trait Variables

##### Athletic identity

Athletic identity was assessed using the Athletic Identity Measurement Scale-Deutsch (AIMS-D; Schmid and Seiler, 2003). AIMS consists of seven items with three subscales: social identity (3 item, e.g., “I consider myself an athlete.”; α = 0.769); exclusivity (2 items, e.g., “Sport is the most important part of my life.”; α = 0.819); and negative affectivity (2 items, e.g., “I feel bad about myself when I do poorly in sport.”; α = 0.785). On a Likert-scale ranging from 1 = *strongly disagree* to 7 = *strongly agree*, athletes were asked to identify how strongly they agreed with the statement. The total score for athletic identity (α = 0.890) is calculated as the sum of all items.

##### Anxiety

Trait Anxiety was assessed using the short form of the State-Trait-Anxiety Inventory (STAI; Grimm, 2009). This measure comprises 10 items on a Likert-scale ranging from 1 = *never* to 4 = *almost always*. A sum score of all items is used to describe trait anxiety (α = 0.807; example item: “I am inclined to take things hard.”).

##### Happiness

Happiness was measured using the short form of the Orientation to Happiness Questionnaire (OTH; Ruch et al., 2014), which consists of three subscales with three items per subscale. Participants are asked to rate their life of pleasure (α = 0.727; e.g., “Life is too short to postpone the pleasures it can provide.”), life of engagement (α = 0.650; e.g., “Regardless of what I am doing, time passes very quickly.”), and life of meaning (α = 0.674; e.g., “My life serves a higher purpose.”) on a 5-point Likert scale ranging from 1 = *very much unlike me* to 5 = *very much like me*.

##### Volitional components

The Volitional Components Questionnaire in Sport (VCQ-SPORT; Elbe and Wenhold, 2005) comprises 60 items, rated on a 4-point scale ranging from 0 = *not true for me at all* to 3 = *exactly true for me*. A total of 23 individual subscales can be organized into 4 more general subscales (lack of activation, self-optimization, loss of focus, and self-impediment).

In this study, we chose 11 items that fit one subcategory of the general subscales: Postponing training (α = 0.696; sample item: “I do not begin a strenuous training until the last minute”) as part of lack of activation; goal setting general (α = 0.699; sample item: “I often focus on what I want to achieve in my sport”) as part of self-optimization; lack of concentration (α = 0.648; sample item: “During training my thoughts often drift away from things I want to concentrate on”) as part of loss of focus; and negative emotionality general (α = 0.652; sample item: “If I get into a bad mood during sport, it is difficult for me to brighten up again”) as part of self-impediment.

##### Coping

To assess different coping strategies, we used the German short version of the Coping Inventory for Stressful Situations (CISS; Kälin, 1995) with 24 items, rated on a 5-point scale ranging from 1 = *not at all* to 5 = *very much*. The questionnaire comprises three subscales with eight items each: task orientation (α = 0.843; e.g., “Go out for a snack or meal.”), emotion orientation (α = 0.752; e.g., “Become very tense.”), and distraction orientation (α = 0.744). Furthermore, distraction orientation can be divided into distraction (α = 0.535; e.g., “Think about how I have solved similar problems.”), and social distraction (α = 0.843; e.g., “Window shop.”).

##### Personality

Based on the Big Five-Model, the Big Five Inventory 10 (BFI-10; Rammstedt et al., 2013) was used. Participants responded on a 5-point Likert scale ranging from 1 = *disagree strongly* to 5 = *agree strongly*. The inventory includes 10 items distributed equally across the five personality dimensions: Extraversion (α = 0.759; e.g., “I see myself as someone who is reserved.”), agreeableness (α = 0.057; e.g., “I see myself as someone who tends to find fault with others.”), conscientiousness (α = 0.550; sample item: “I see myself as someone who tends to be lazy”), neuroticism (α = 0.647; sample item: “I see myself as someone who gets nervous easily”), and openness (α = 0.373; sample item: “I see myself as someone who has few artistic interests”).

#### Psychological State Variables

##### Discrete emotions (State)

The Discrete Emotions Questionnaire (DEQ; Harmon-Jones et al., 2016) was translated into German with the help of four bilingual speakers. The DEQ consist of 32 items (i.e., single words as “calm,” or “lonely”) and 8 subscales (i.e., anger: α = 0.733; disgust: α = 0.807; fear: α = 0.786; anxiety: α = 0.726; sadness: α = 0.790; desire: α = 0.716; relaxation: α = 0.869; happiness: α = 0.828). Participants indicated how they currently felt using a Likert scale from 1 = *not at all* to 7 = *an extreme amount*.

##### Stress (State)

The short version of the Perceived Stress Scale (PSS-10; Klein et al., 2016) was used to assess the perceived level of stress within the last 4 weeks. The PSS includes the two subscales perceived helplessness (α = 0.854, e.g., “In the last month, how often have you felt nervous and “stressed”?”) and self-efficacy (α = 0.723, e.g., “In the last month, how often have you felt that things were going your way?”). The total score of perceived stress (α = 0.882) is the sum of all perceived helplessness items and reversed perceived self-efficacy items.

##### Open questions on problems, coping strategies, wanted and received external support, and joyful anticipation

Due to the exploratory nature of the study, we decided to include open questions. In detail, questions assessing problems included: “Please list your three biggest challenges and problems due to the current situation (COVID-19 pandemic).”; for coping strategies: Please list the three most important strategies you use to deal with and overcome your current challenges and problems; for received external support: “Please list the most important help and support you receive in the current situation (COVID-19 pandemic), e.g., from coaches, association, family or friends.”; for wanted external support: “Please list three additional concrete types of help and support you would like to receive and from whom you would like to receive it.”; and finally for joyful anticipation: “List three things you are most looking forward to following the COVID-19 pandemic.”

### Data Analysis

Data were first checked for outliers and normal distribution. There were four outliers on the DEQ disgust subscale. Data were not normally distributed for most variables. However, it can be expected that the data represent the actual mean of a variable due to sample size (Bortz and Döring, 2006). Therefore, all analyses were performed using parametric statistics.

Mean differences between groups were tested by running several one-way analyses of variance (ANOVA). Significant main effects were followed up by Bonferroni-adjusted *post hoc* tests. We chose more open *post hoc* tests, for example in comparison to planned comparisons, because we aimed to capture as much information as possible due to the novelty of the research topic. Differences between groups related to gender and type of sport were performed using Chi-Square tests. For all analyses the level of significance was initially set at *p* > 0.05.

Finally, participants’ qualitative responses (i.e., problems, coping strategies, wanted and received external support, and joyful anticipation) were exported to an Excel spreadsheet. After two researchers familiarized themselves with the data (Braun et al., 2016), they inductively and independently coded the data into tentative categories based on their own reflections. These categories were then discussed by both researchers (Creswell et al., 2007) and disagreements were resolved by discussion. These resulting categories, including the participants’ mentioning each category, were counted and then listed for all participants based on their respective motivation to train and exercise (i.e., less vs. more motivated vs. no change).

## Results

Table 3 lists all descriptive statistics for psychological trait variables and Table 4 for psychological state variables. Table 5 features a correlation matrix for all variables.

**TABLE 3:** Descriptive statistics of trait variables (i.e., athletic identity, trait anxiety, orientation to happiness, volitional competencies, coping style, BIG 5) for the complete sample (*N* = 95) dependent on motivational change to exercise (less: *n* = 33; same: *n* = 39; more: *n* = 23).

**Change in motivation to exercise**	***M***	***SD***	***Min***	***Max***
**Athletic identity**				
Less	50.79	11.59	16	67
Same	53.72	7.79	34	65
More	46.66	11.40	18	63
Total	51.00	10.40	16	67
**Trait anxiety**				
Less	33.18	5.23	23	44
Same	30.51	4.19	24	40
More	31.74	4.92	25	48
Total	31.74	4.84	23	48

**Orientation to Happiness**				

**Pleasure**				
Less	3.97	0.53	3.00	5.00
Same	3.90	0.72	2.00	5.00
More	4.10	0.67	2.00	5.00
Total	3.98	0.65	2.00	5.00
**Engagement**				
Less	3.17	0.57	2.33	4.33
Same	3.34	0.66	1.67	4.67
More	3.30	0.71	2.00	5.00
Total	3.27	0.63	1.67	5.00
**Meaning**				
Less	2.90	0.82	1.00	4.33
Same	3.27	0.79	1.00	4.67
More	2.64	0.99	1.00	5.00
Total	2.99	0.88	1.00	5.00

**Volitional components**				

**Postponing training**				
Less	4.79	1.65	3.0	9.0
Same	3.85	1.06	3.0	7.0
More	4.57	1.70	3.0	8.0
Total	4.35	1.50	3.0	9.0
**Goal setting**				
Less	8.06	1.87	4.0	11.0
Same	8.69	1.58	6.0	12.0
More	8.04	2.08	3.0	12.0
Total	8.32	1.82	3.0	12.0
**Lack of concentration**				
Less	3.18	1.29	2.0	7.0
Same	3.46	1.27	2.0	6.0
More	3.87	1.29	2.0	6.0
Total	3.46	1.29	2.0	7.0
**Negative emotionality**				
Less	6.03	2.23	3.0	11.0
Same	5.20	1.66	3.0	10.0
More	5.22	1.65	3.0	9.0
Total	5.49	1.90	3.0	11.0

**Coping styles**				

**Emotion-oriented coping**				
Less	2.95	0.85	1.38	4.50
Same	2.66	0.5	1.50	3.88
More	2.82	0.5	1.75	4.00
Total	2.80	0.65	1.38	4.50
**Task-oriented coping**				
Less	3.54	0.66	2.13	4.88
Same	3.67	0.59	2.13	4.75
More	3.55	0.80	1.00	4.50
Total	3.60	0.67	1.00	4.88
**Distraction-oriented coping**				
Less	2.77	0.67	1.00	4.13
Same	2.68	0.65	1.63	4.38
More	2.78	0.78	1.00	3.88
Total	2.74	0.68	1.00	4.38

**Big Five Personality traits**				

**Extraversion**				
Less	3.45	1.15	1.5	5.0
Same	3.20	1.07	1.5	5.0
More	3.78	0.64	2.0	5.0
Total	3.43	1.03	1.5	5.0
**Agreeableness**				
Less	3.26	0.87	2.0	5.0
Same	3.42	0.62	2.5	5.0
More	3.28	0.77	1.0	4.5
Total	3.33	0.75	1.0	5.0
**Conscientiousness**				
Less	3.52	0.86	2.0	5.0
Same	3.62	0.77	2.0	5.0
More	3.57	0.82	2.5	5.0
Total	3.57	0.80	2.0	5.0
**Neuroticism**				
Less	2.94	0.95	1.0	4.5
Same	2.61	0.90	1.0	4.5
More	2.72	0.99	1.5	5.0
Total	2.75	0.94	1.0	5.0
**Openness**				
Less	3.59	0.96	1.5	5.0
Same	3.12	0.80	2.0	5.0
More	3.43	1.00	1.0	5.0
Total	3.36	0.92	1.0	5.0

**TABLE 4:** Descriptive statistics of state variables (i.e., discrete emotions, perceived stress) for the complete sample (*N* = 95) dependent on motivational change to exercise (less: *n* = 33; same: *n* = 39; more: *n* = 23).

**Change in motivation to exercise**	***M***	***SD***	***Min***	***Max***
**Discrete emotions**				
**Anger (state)**				
Less	3.14	1.15	1.50	5.75
Same	2.42	1.01	1.00	6.00
More	2.43	0.93	1.00	4.25
Total	2.67	1.09	1.00	6.00
**Disgust (state)**				
Less	1.36	0.63	1.00	3.50
Same	1.49	0.84	1.00	4.00
More	1.32	0.62	1.00	3.50
Total	1.40	0.72	1.00	4.00
**Fear (state)**				
Less	2.06	0.95	1.00	4.75
Same	1.85	1.10	1.00	5.00
More	1.57	0.74	1.00	3.75
Total	1.85	0.98	1.00	5.00
**Anxiety (state)**				
Less	2.88	1.15	1.00	5.50
Same	2.38	1.02	1.00	4.75
More	2.53	1.04	1.00	5.25
Total	2.59	1.08	1.00	5.50
**Sadness (state)**				
Less	3.55	1.30	1.50	6.75
Same	2.78	1.25	1.00	6.00
More	2.88	1.05	1.25	5.00
Total	3.07	1.26	1.00	6.75
**Desire (state)**				
Less	3.73	1.15	1.75	6.00
Same	3.96	1.32	1.50	6.25
More	3.80	1.13	1.50	5.75
Total	3.84	1.21	1.50	6.25
**Relaxation (state)**				
Less	3.39	1.11	1.25	6.75
Same	4.15	1.25	1.00	6.50
More	4.29	1.42	1.25	6.25
Total	3.92	1.30	1.00	6.75
**Happiness (state)**				
Less	3.23	1.29	1.00	6.25
Same	3.86	1.21	1.25	6.00
More	4.05	1.12	2.25	6.50
Total	3.69	1.25	1.00	6.50
**Perceive stress**				
**Helplessness**				
Less	12.70	4.55	2.0	21.0
Same	9.54	4.54	3.0	20.0
More	11.22	5.28	2.0	21.0
Total	11.04	4.88	2.0	21.0
**Self-efficacy**				
Less	8.00	2.75	2.0	14.0
Same	9.31	2.58	5.0	13.0
More	8.91	2.29	5.0	14.0
Total	8.76	2.61	2.0	14.0
**Total stress**				
Less	20.69	6.71	5.0	32.0
Same	16.23	6.57	6.0	31.0
More	18.30	7.14	6.0	31.0
Total	18.28	6.97	5.0	32.0

**TABLE 5:** Correlation matrix of psychological trait and state variables.

**Variable**	**1**	**2**	**3**	**4**	**5**	**6**	**7**	**8**	**9**	**10**	**11**	**12**	**13**	**14**	**15**	**16**	**17**	**18**	**19**	**20**	**21**	**22**	**23**	**24**	**25**	**26**	**27**	**28**	**29**	**30**	**31**	**32**	**33**	**34**
1. Age	—																																	
2. Exclusivity (AIMS)	0.02	—																																
3. Negative affectivity (AIMS)	−0.16	0.62**	—																															
4. Social identity (AIMS)	0.00	0.62**	0.68**	—																														
5. Athletic identity total (AIMS)	−0.09	0.86**	0.83**	0.85**	—																													
6. Trait anxiety	−0.14	0.10	0.09	0.02	0.11	—																												
7. Pleasure (OTH)	−0.04	−0.02	0.00	0.02	−0.43	−0.23*	—																											
8. Engagement (OTH)	0.02	0.05	0.19	0.07	0.10	−0.36**	0.24*	—																										
9. Meaning (OTH)	0.08	0.06	0.14	0.10	0.05	−0.39**	0.04	0.40**	—																									
10. Postponing training (VCQ-SPORT)	0.00	0.06	−0.17	−0.07	−0.02	0.16	0.01	−0.12	−0.18	—																								
11. Goal setting (VCQ-SPORT)	−0.20	0.51**	0.53**	0.47**	0.58**	−0.03	0.03	0.28**	0.12	−0.05	—																							
12. Lack of conentration (VCQ-SPORT)	0.00	−0.01	−0.09	−0.09	−0.02	0.25*	−0.05	−0.13	−0.13	0.19	−0.05	—																						
13. Negative emotionality (VCQ-SPORT)	−0.02	0.15	0.02	0.09	0.16	0.31**	−0.05	−0.09	−0.12	0.21*	0.05	0.20	—																					
14. Task-oriented coping (CISS)	0.05	−0.06	0.12	0.02	−0.01	−0.56**	0.16	0.38**	0.36**	−0.21*	0.14	−0.28**	−0.11	—																				
15. Emotion-oriented coping (CISS)	−0.31**	0.17	0.16	0.18	−0.24*	0.52**	0.02	−0.17	−0.20*	0.20	0.07	0.23*	0.43	−0.38**	—																			
16. Discration-oriented coping (CISS)	0.02	−0.04	0.03	−0.06	−0.04	−0.25*	0.35**	0.29**	0.29**	0.06	−0.12	0.05	−0.20	0.24*	−0.11	—																		
17. Distraction-oriented coping: Distration (CISS)	0.07	0.08	0.08	−0.01	0.06	−0.12	0.17	0.23*	0.14	0.12	−0.10	0.10	−0.03	0.15	−0.10	0.72**	—																	
18. Distraction-oriented coping: Social (CISS)	−0.02	−0.11	−0.02	−0.08	−0.09	−0.26*	0.36**	0.25*	0.30**	0.00	−0.10	−0.01	−0.25	0.23*	−0.08	0.87**	0.29**	—																
19. Extraversion (BFI-10)	0.03	−0.16	−0.12	−0.11	−0.17	−0.34**	0.32**	0.12	0.13	0.02	−0.07	−0.02	−0.18	0.20	−0.22	0.47**	0.18	0.52**	—															
20. Agreeableness (BFI-10)	0.00	0.00	0.01	0.03	0.00	−0.34**	0.22*	0.25*	0.23*	−0.09	0.05	−0.09	−0.12	0.16	−0.12	0.45**	0.26*	0.44**	0.27**	—														
21. Conscientiousness (BFI-10)	0.10	0.22*	0.42**	0.21*	0.27**	0.02	−0.05	0.19	0.10	−0.35**	0.20	0.07	−0.11	0.13	0.06	−0.02	−0.08	0.03	−0.02	−0.07	—													
22. Neuroticism (BFI-10)	−0.14	0.05	0.05	0.03	0.06	0.56**	0.02	−0.19	−0.14	0.03	−0.01	0.27**	0.24	−0.39**	0.60	−0.03	−0.09	0.02	−0.12	−0.09	0.31**	—												
23. Openness (BFI-10)	−0.07	−0.14	−0.15	−0.14	−0.16	0.19	0.13	0.03	0.04	0.05	−0.07	−0.04	0.13	−0.15	0.13	0.26*	0.26*	0.18	0.09	0.00	−0.05	0.34**	—											
24. Anger (DEQ)	0.08	0.37	0.44	0.31	−0.09	0.33**	−0.13	−0.12	−0.18	0.09	−0.08	0.23*	0.28**	0.02	0.08	−0.07	−0.05	−0.06	−0.03	−0.02	0.02	0.17	−0.04	—										
25. Disgust (DEQ)	0.07	0.13	0.03	−0.02	0.05	0.13	−0.07	−0.09	0.02	0.16	0.07	0.14	0.15	0.05	0.01	0.06	0.03	0.07	−0.10	−0.11	−0.09	0.06	0.01	0.35**	—									
26. Fear (DEQ)	0.15	0.22*	0.13	0.08	0.19	0.36**	−0.15	−0.19	−0.13	0.21*	0.03	0.11	0.24*	−0.06	0.13	0.04	0.09	−0.01	−0.07	−0.09	0.00	0.16	−0.10	0.54**	0.54**	—								
27. Anxiety (DEQ)	0.06	0.12	0.05	0.04	0.13	0.43**	−0.20	−0.19	−0.21*	0.16	0.06	0.10	0.32**	−0.05	0.20	−0.10	−0.02	−0.13	−0.13	−0.30	0.02	0.27**	0.04	0.58**	0.37**	0.78**	—							
28. Sadness (DEQ)	−0.17	0.04	0.02	−0.04	0.04	0.54**	−0.06	−0.23*	−0.24*	0.09	−0.16	0.06	0.22*	−0.18	0.24*	−0.13	−0.12	−0.09	−0.14	−0.08	−0.07	0.25*	−0.03	0.59**	0.13	0.53**	0.53*	—						
29. Desire (DEQ)	−0.03	0.03	−0.04	0.03	0.04	0.07	0.12	−0.14	0.04	0.03	0.01	0.06	0.17	0.05	−0.03	0.04	0.06	0.02	−0.01	0.03	0.01	0.01	−0.05	0.28**	0.13	0.20	0.21	0.26*	—					
30. Relaxation (DEQ)	−0.08	−0.05	−0.07	−0.05	−0.12	−0.32**	0.18	0.03	0.18	−0.05	0.10	−0.08	−0.44**	0.04	−0.24*	0.04	0.01	0.05	0.03	−0.02	−0.12	−0.30**	−0.05	−0.48**	0.05	−0.33**	−0.43**	−0.40**	0.00	—				
31. Happiness (DEQ)	0.14	−0.11	−0.05	−0.07	−0.16	−0.25*	0.18	0.03	0.14	−0.07	0.04	0.00	−0.29**	0.03	−0.14	0.06	0.04	0.06	0.00	−0.16	0.07	−0.14	0.03	−0.32**	0.14	−0.12	−0.22*	−0.44**	0.00	0.63	—			
32. Helplessness (PSS-10)	−0.10	−0.01	−0.01	−0.13	−0.02	0.52**	0.02	−0.21*	−0.36**	0.20*	−0.10	0.18	0.30**	−0.23*	0.38**	0.00	−0.05	0.03	0.06	−0.11	0.01	0.40**	0.09	0.56**	0.14	0.57**	0.60**	0.56**	0.221*	−0.54	−0.31**	—		
33. Self-efficacy (PSS-10)	0.13	0.02	−0.02	0.06	0.01	−0.51**	−0.04	0.19	0.26*	−0.06	0.06	−0.19	−0.26*	0.20	−0.28**	0.03	0.06	0.00	−0.09	−0.11	0.12	−0.30**	−0.04	−0.51**	−0.01	−0.32**	−0.37**	−0.59**	−0.14	0.50	0.47**	−0.70**	—	
34. Perceived Stress (PSS-10)	−0.12	−0.02	0.00	−0.11	−0.02	0.56**	0.03	−0.22*	−0.35**	0.16	−0.09	0.20	0.31**	−0.23*	0.37**	−0.01	−0.06	0.02	0.07	−0.04	−0.04	0.39**	0.08	0.58**	0.10	0.52**	0.56**	0.62**	0.21*	−0.56	−0.39**	0.96**	−0.87**	—

*p < 0.05. **p < 0.01; AIMS, Athletic Identity Measurement; OTH, Orientation to Happiness; VCQ-SPORT, Volitional Components Questionnaire in Sport; CISS, Coping Inventory for Stressful Situations; BFI-10, Big Five Inventory 10; DEQ, Discrete Emotions Questionnaire; PSS-10, Perceived Stress Scale.

### Differences in Demographic and Sport Specific Variables

Age did not significantly differ between athletes with different self-reported motivation, *F*(2,92) = 1.11, *p* = 0.334. However, significantly more female athletes were less motivated to train (*n* = 23) during the corona crisis, χ^2^ (2, *N* = 95) = 7.11, *p* = 0.029, ω = 0.274 than male athletes (*n* = 10).

There was no significant difference in athletes’ motivation related to the type of sport they practice, χ^2^ (4, *N* = 92) = 1.54, *p* = 0.82. However, the one-way ANOVA showed a significant difference in training hours prior to the lockdown with respect to athletes’ motivation, *F*(2,92) = 4.47, *p* = 0.014. Bonferroni-adjusted *post hoc* tests showed a higher number of training hours during normal circumstances in athletes who reported no change in motivation in comparison to athletes more motivated to train during lockdown, *p* = 0.015, *d* = 0.93. Finally, there was no difference in the number of competitions, *F*(2,92) = 1.81, *p* = 0.170.

### Differences in Psychological Trait Variables

Athletic identity significantly differed depending on athletes’ self-reported motivation to train during the COVID-19 crisis, *F*(2,92) = 3.35, *p* = 0.035. *Post hoc* tests showed that athletes who were more motivated to train, showed lower athletic identity than athletes who reported no change in motivation to exercise during the lockdown, *p* = 0.03, *d* = 0.72, 95% CI [–13.53, –0.52].

Trait anxiety showed a tendency, *F*(2,92) = 3.35, *p* = 0.053. Due to the exploratory nature of the study, we decided to investigate the *post hoc* tests (e.g., Lautenbach et al., 2016). We found a tendency for higher trait anxiety in athletes less motivated to train in comparison to athletes who reported no change in motivation to train, *p* = 0.059, *d* = 0.53, 95% CI [–0.72, 5.41].

Meaning, as a subscale of the OTH differed significantly between groups, *F*(2,92) = 4.16, *p* = 0.019, whereas pleasure (*p* = 0.519) and engagement (*p* = 0.518) showed no differences. *Post hoc* tests revealed that meaning was significantly less pronounced in athletes who reported to be more motivated to train during the lockdown, *p* = 0.018, *d* = 0.75, 95% CI [–1.17, –0.08].

Volitional components did not differ with respect to the subscales general goal setting (part of self-optimization; *p* = 0.244), lack of concentration (part of loss of focus; *p* = 0.148), and general negative emotionality (part of self-impediment; *p* = 0.133). However, the subscale postponing training (part of lack of energy), *F*(2,92) = 4.1, *p* = 0.02, was significantly lower in athletes who reported no change in motivation to train compared to athletes who were less motivated, *p* = 0.022, *d* = 0.68, 95% CI [–1.78, –0.10].

Coping strategies did not differ significantly between athletes (task orientation: *p* = 0.676; emotion orientation: *p* = 0.157; distraction orientation distraction: *p* = 0.927; distraction orientation social: *p* = 0.833; distraction orientation total: *p* = 0.816).

Personality parameters did not differ significantly between athletes who were less or more motivated to train or reported no change in motivation to train (extraversion: *p* = 0.1; agreeableness: *p* = 0.608; conscientiousness: *p* = 0.84; neuroticism: *p* = 0.342; openness: *p* = 0.083).

### Differences in Psychological State Variables

Discrete emotions differed significantly between groups, anger: *F*(2,92) = 4.97, *p* = 0.009; sadness: *F*(2,92) = 3.99, *p* = 0.022; relaxation: *F*(2,92) = 4.72, *p* = 0.011; happiness: *F*(2,92) = 3.71, *p* = 0.028. *Post hoc* tests showed that anger is significantly higher in athletes who are less motivated to train in comparison to athletes who reported no changes, *p* = 0.014, *d* = 0.67, 95% CI [0.11, 1.32], and athletes who were more motivated, *p* = 0.045, *d* = 0.67, 95% CI [0.01, 1.39]. Sadness was significantly higher in athletes who were less motivated to train in comparison to those that reported no change, *p* = 0.025, *d* = 0.63, 95% CI [0.07, 1.48]. Athletes that were less motivated to train felt less relaxed in comparison to athletes that reported no change in motivation, *p* = 0.033, *d* = 0.66, 95% CI [–1.49, –0.05], and in comparison to athletes that reported to be more motivated, *p* = 0.027, *d* = 0.74, 95% CI [–1.74, –0.08]. Finally, athletes who were less motivated to train reported to be less happy than athletes who were more motivated to train, *p* = 0.045, *d* = 0.61, 95% CI [–1.62, –0.01].

Perceived stress level within the previous 4 weeks differed significantly the groups, *F*(2,92) = 3.901, *p* = 0.024. *Post hoc* analyses showed a significantly higher stress level in athletes who had less motivation to exercise in comparison to athletes who had no change in motivation, *p* = 0.006, *d* = 0.68, 95% CI [0.57, 8.37]. Also a significant difference in perceived helplessness was detected between groups, *F*(2,92) = 4.002, *p* = 0.022, showing that athletes with less motivation to train during the COVID-19 lockdown compared to athletes who experienced no change in motivation had higher levels of helplessness, *p* = 0.006, *d* = 0.71, 95% CI [0.43, 5.89]. No significant differences were found regarding self-efficacy, *F*(2,92) = 2.360, *p* = 0.1.

### Correlations Between Psychological Trait and State Variables

Independent of our specific hypotheses and for purposes of future interest, Table 5 presents a correlation matrix of psychological trait and state variables.

### Detailed Data on Problems, Coping Strategies, Wanted and Received External Support, and Joyful Anticipation

In total, 270 problems (*n*_*less*_ = 96; *n*_*same*_ = 111; *n*_*more*_ = 63) were mentioned by the athletes (see Supplementary Table 2 for details). The most often mentioned problems and challenges during lockdown were closed sports facilities (*n* = 62; 64%), followed by missing social contacts (*n* = 35; 37%), staying in shape (*n* = 25; 26%), issues concerning motivation (*n* = 23; 24%) and the missing social contact in the sport context (e.g., teammates; *n* = 19; 20%). With respect to the groups (i.e., less vs. more motivated vs. no change) however, the distribution differs on a descriptive level. Less motivated athletes reported lack of motivation more often (*n* = 16; 48%) than athletes who report no change in motivation (*n* = 5, 13%) or who were more motivated (*n* = 2^1^; 9%). In addition, missing social contacts was mentioned less often by motivated athletes (*n* = 8; 24%) in comparison to the two other groups (*n*_*same*_ = 19; 49%; *n*_*more*_ = 8; 35%). Finally, athletes who are less motivated were less concerned about closed sports facilities (*n*_*less*_ = 15; 45%; *n*_*same*_ = 30; 77%; *n*_*more*_ = 16; 70%).

In the category coping strategies, athletes reported a total of 268 responses (see Supplementary Table 3 for details). The most often mentioned coping strategies were related to training and exercise online (*n* = 63; 97%), organization and structure (e.g., structuring the day; *n* = 44; 61%), using online tools and software to maintain social contact (*n* = 29; 44%) and finding alternative sports or ways to train and exercise (e.g., skating, weightlifting; *n* = 20; 33%). Again, with respect to the groups (i.e., less vs. more motivated vs. no change) however, the distribution of mentioned strategies differed on a descriptive level. Athletes who were less motivated to train mentioned the use of online training less often (*n*_*less*_ = 11; 33%; *n*_*same*_ = 30; 77%; *n*_*more*_ = 22; 96%).

In the category received external support, a total of 122 responses (*n*_*less*_ = 45; *n*_*same*_ = 51; *n*_*more*_ = 26) were provided by the athletes (see Supplementary Table 4 for details). The most frequently mentioned received external support was from family (*n* = 40; 42%), friends (*n* = 24; 25%), significant others (*n* = 20; 21%), and coach (*n* = 15; 16%).

In the category of wanted external support, a total of 94 replies (*n*_*less*_ = 35; *n*_*same*_ = 24; *n*_*more*_ = 35) were provided by the athletes (see Supplementary Table 5 for details). The most frequently mentioned desired external support was by coaches (*n* = 21; 22%), the state (*n* = 20; 21%), and the university (*n* = 13; 14%). Finally, 15 athletes (16%) stated that they need no further support.

In the category of joyful anticipation, a total of 220 responses (*n*_*less*_ = 97; *n*_*same*_ = 57; *n*_*more*_ = 65) were provided by the athletes (see Supplementary Table 6 for details). Athletes who were less motivated to train responded the most. Overall, the most often mentioned joyful anticipation items were social contacts (*n* = 58; 61%), leisure activities (e.g., sitting in a café or restaurant, partying; *n* = 40; 42%), practicing sport with others (*n* = 25; 26%), returning to normal training (*n* = 25; 26%), and open sports facilities (*n* = 24; 25%). On a descriptive level there were, however, differences between groups (i.e., less vs. more motivated vs. no change). Social contacts were mentioned less often by athletes who did not report a change in motivation to train during the lockdown in comparison to those who were more or less motivated (*n*_*less*_ = 29; 88%; *n*_*same*_ = 7; 17%; *n*_*more*_ = 22; 96%).

The Supplementary Tables 2–6 show all mentions for all categories.

## Discussion

Exercising and training is highly important especially during lockdown (Brand et al., 2020; González-Valero et al., 2020; Maher et al., 2020; Mehrsafar et al., 2020). Thus, the aim of the study was to identify athletes who experienced a change in motivation to exercise due to the lockdown. In detail, we aimed to more precisely understand who (i.e., demographic, sport-specific and psychological trait and state variables) experienced a change in motivation. Further, we aimed to explore the problems they experienced and what they did to help themselves, what support they received from others, what support they missed and what they were looking forward to after the lockdown. In doing so, we used a combined approach of open and closed questions to provide in-depth information on the German amateur and recreational athletes’ psychological state.

Overall our results show that 41% of amateur and recreational athletes reported no change in motivation to exercise during the lockdown, whereas 35% stated they were less motivated and 24% stated an increase in motivation to train. Although we only assessed motivation to train and not self-reported exercise frequency, our results are comparable to Brand et al. (2020); 44% no change, 24% decrease, 32% increase) as well as to Mutz and Gerke (2020) with respect to individuals who maintained their level of physical activity (42% no change). Especially from a theoretical perspective our results were unexpected as they are not necessary in line with SDT (Deci and Ryan, 2000). In our data, however, 35% of amateur and recreational athletes felt less motivated to exercise; as motivation is related to action (e.g., Heckhausen and Heckhausen, 2008), we would argue that this number is alarming in terms of maintaining athletes’ physical and mental health (Mehrsafar et al., 2020). On the other hand, our data show that one quarter of the athletes state an increase in motivation to exercise. At first glance, this may be considered a positive development, especially with regard to the general population (Brand et al., 2020). However, athletes who experienced an increase in motivation showed similar values in trait and state variables as athletes that reported a decrease in motivation. Thus, this increased motivation to exercise might indicate that it has a compensatory function, which may not necessarily be considered positive.

### Motivational Differences With Regard to Demographic and Sport Specific Variables

Overall, we found no differences in age, type of sport, and number of competitions per year between the groups (less vs. same vs. more motivation to exercise). However, significant differences were found for gender distribution and amount of exercise prior to COVID-19.

In detail and not in line with our hypothesis, age does not differ between the three groups. We argue that this might be due to more encounters with stressors and lower stress levels in older individuals in the general population (Klein et al., 2016). However, not only did we not detect significant correlations between age and perceived stress (see correlation matrix in the Supplementary Material). Specifically, the lockdown and COVID-19 crisis in general presents the new factors uncertainty and lack of control (Clemente-Suárez et al., 2020; Leisterer et al., under review) such that additional life experience does not impact stress perception and thus, motivation (see PSI theory) in our sample. In other words, effects of the lockdown on motivation seem to be irrespective of the amateur and recreational athletes’ age. In line with previous research (di Fronso et al., 2020), the type of sport (team vs. individual) does not make a difference with respect to motivation to exercise. In addition to the idea that we should focus on the way the sport is trained (individually vs. with a team; see AASP (Association for Applied Sport Psychology) Blog, 2020), we would argue that athletes who can train individually are less affected by hygiene restrictions in training than those who normally train in teams but have to distance socially during the pandemic. Finally, contrary to our expectations, no difference in the number of competitions prior to lockdown was detected between groups. Athletes prepare for competitions via training, and since competitions were postponed or canceled, we assumed that motivation would decrease especially in athletes with typically more competitive encounters. Future research should assess the importance of competitions as well as athletes’ goals in order to understand the impact of competitions on motivation to exercise.

We found differences between groups with respect to gender distribution and training hours prior to COVID-19. In accordance with our hypothesis and in accordance with previous research (di Fronso et al., 2020), we found that more female athletes were less motivated to train in comparison to males. In our sample, female athletes showed higher levels of stress (*p* = 0.029, *d* = 0.46) and lower levels of relaxation (*p* = 0.012, *d* = 0.57). These medium effects might be explained by the finding that female negative emotions and feelings are often related to avoidance motivation (see Elliot et al., 2013), which in turn might explain why the females are less motivated (see also PSI theory). As expected, a higher number of training hours prior to lockdown were detected in amateur and recreational athletes who reported no change in motivation compared to amateur and recreational athletes who were more motivated to train during the lockdown. Based on the stress-buffer hypothesis, which suggests that better trained individuals cope better with stress due to the availability of increased psychophysiological resources, it could be argued that external stressors such as the corona lockdown result in less psychological and physiological stress in better trained individuals (e.g., Gerber et al., 2018) and therefore have lower impact on their motivation (PSI theory). On the other hand, more motivated athletes may have come to the realization that they have to spend their time differently due to the lockdown, and might in turn become distracted. This line of argument, however, infers causal connections not assessed in this study, and it might also very well be that during concurrent spring season in Germany, people exercised more on average (Pivarnik et al., 2003). Despite our fairly small sample compared to other recent publications on the topic, this effect is large and needs further investigation. In future, not only should motivation be assessed more objectively but motivation to exercise should also be assessed, as this is related to exercise commitment, also during a lockdown (Angosto et al., 2020).

### Motivational Differences With Regard to Psychological Trait Variables

Overall, all trait variables (except lack of concentration as a volitional component) show an interesting but unexpected trend: Highest or lowest values in comparison to amateur and recreational athletes that are less and more motivated to exercise. In other words, we observe a U- or inverted-U-shape. On a descriptive level, amateur and recreational athletes that reported no change in motivation show the highest values in athletic identity, engagement and meaning (i.e., happiness), goal setting (part of volitional component), task-oriented coping, agreeableness and conscientiousness (i.e., personality). On the other hand, these athletes have the lowest values in trait anxiety, pleasure (i.e., happiness), postponement of training and negative emotionality (i.e., volitional competence), emotion- and distraction-oriented coping, extraversion, agreeableness, neuroticism, and openness (i.e., personality).

It could be argued that a combination of these factors might protect athletes from experiencing motivational changes in one or the other direction and thus, could be considered positive in providing stability. In accordance with the very broad concept of Control Balance Theory (Tittles, 1995) this would mean that the possible occurrence of *divergent* or, to be more abstract, different behavior is low when received (i.e., external) and perceived (i.e., internal) control is in balance. Due to a decrease in control due to lockdown restrictions, internal resources or skills to regain control must be especially high to restore balance. In view of our data this means that certain trait variables should be more pronounced in order to keep internal control high. However, future research should further investigate such clusters, as our sample size did not allow for clustering to empirically test this idea. Even though it is interesting to observe this pattern in trait variables that might provide stability and continuity, only some of these variables differ statistically between the three groups.

Significant differences between the groups in psychological trait variables were only found in athletic identity, meaning (i.e., happiness), and postponing training as a volitional competence. In contrast to Costa et al. (2020) and our hypothesis, we found the highest athletic identity in athletes that experienced no change in motivation to exercise. It was significantly higher in those athletes in comparison to athletes that reported an increase in motivation to exercise. Athletic identity in the sample by Costa et al. (2020) was divided according to the median value within the sample and thus, might have led to different results. It can be speculated that in contrast to the idea that athletic identity contributes to athletes’ vulnerability during a crisis (Batey and Parry, 2020), it may be a protective factor in moderating changes in motivation to exercise.

Higher levels in meaning on the orientation to happiness scale indicate a higher cultivation and orientation toward virtues and their use in service of a greater good, which is frequently but not necessarily of a religious nature (Peterson et al., 2007). Meaning is positively related to subjective well-being which is mediated via prosocial behavior and goal-directed behavior (for more details see e.g., Yang et al., 2017). Thus, individuals actively seek out chances to pursue their goals, which may explain the finding that athletes high in meaning, experience no change in motivation to exercise. Interestingly, we found significant positive correlations between meaning and task-oriented coping (*p* < 0.001, *r* = 0.38). A significantly higher level in meaning has only been detected in athletes who report no change in motivation to exercise compared to athletes with increased motivation. On the one hand this finding contradicts comparable research that detects a positive relation between meaning and motivation at work (Leite, 2018) and on the other, this might suggest that sport is used as compensation for meaning in life for athletes who increased their motivation to exercise. While this is arguably highly speculative, it should not be overlooked. In comparison, athletes who reported an increase in motivation to exercise showed lower levels in athletic identity compared to those reporting no change in motivation. It could be argued that athletes who report no change in motivation to exercise might have shifted their meaning toward their training to adapt to the pandemic. This idea is in line with the argument that the COVID-19 pandemic is such a drastic event that trait-like variables such as athletic identity can be affected (Costa et al., 2020, p. 2). However, due to our cross-sectional design, we cannot further investigate this potentially bidirectional link between meaning and athletic identity. Overall, again we argue that higher levels of meaning could help to stay balanced during situations of crisis (i.e., COVID-19 pandemic) as they foster task-oriented coping and goal-directed behavior.

In a similar line of reasoning, athletes who reported no change in motivation show significantly lower levels of postponing training in comparison to athletes who are less motivated, thereby reporting the lowest lack of energy as one volitional component. This is in accordance to our hypothesis. In addition, it underlines the theoretical assumption that volitional components help individuals to pursue their goals and protect against external disruptions or stressors (PSI theory). However, future research should further explore this particular idea as our data only allow speculation on causal relationships.

### Motivational Differences With Regard to Emotions and Stress

Overall, we found that the pattern of state variables is similar to that of trait variables (except relaxation and happiness). The highest values in positive emotions and self-efficacy as well as the lowest values in negative emotions, helplessness and perceived stress can be seen in athletes that reported no change in motivation to exercise. Thus, it can be assumed that these athletes are least affected by the COVID-19 pandemic. Interestingly, in comparison to norm values of the general population (age group 20–29: *M_*helplessness*_* = 7.6; *M_*self*__–efficacy_* = 5.8; *M_*perceived stress*_* = 12.74) amateur and recreational athletes, also those who report no change in motivation, show higher levels in helplessness, perceived stress but also in self-efficacy. The descriptively higher level of perceived stress in comparison to norm values is in line with results from Italian athletes that showed an increase in stress pre- to post-corona lockdown (di Fronso et al., 2020). Successful athletes are known to have high self-efficacy, whereas lack of self-efficacy is related to failure (e.g., Feltz, 2007). Athletes also have generally higher levels of self-efficacy compared to the general population (e.g., Laborde et al., 2016). Thus, it can be considered positive that high levels of self-efficacy are present in athletes even in times of crisis. Based on our data on stress and even in contrast to Iancheva et al. (2020) as well as to Makarowski et al. (2020) who assessed data fairly late after the lockdown, and in line with di Fronso et al. (2020), we argue that the COVID-19 pandemic and lockdown increased athletes’ stress levels.

Comparing the different groups (less vs. same vs. more motivation to exercise), we found expected differences in anger, sadness, relaxation, and happiness. In detail, less motivated athletes showed higher levels of anger and sadness compared to athletes who experienced no change in motivation. Additionally, they experience more anger in comparison to athletes who reported more motivation. Anger is known to occur after failure for example due to externally stable difficult tasks, which may eventually have a negative impact on future motivation (Weiner, 1985). For our data this might mean that athletes feel anger and thus, are less motivated. This causal relation, however, is speculative and needs to be investigated in longitudinal studies. The function of sadness on the other hand is to withdraw, heal, seek help, and rebuild energy and formulate new goals (Scarantion, 2018). This can be seen in our data as well. The sadder that athletes feel, the less relaxed (*p* < 0.001, *r* = −0.4), the more helpless (*p* < 0.001, *r* = 0.56), and the more stressed (*p* < 0.001, *r* = 0.62) they feel.

Relaxation and happiness were significantly lower in less motivated compared to more motivated athletes. In addition, relaxation was significantly lower in athletes who had a decrease in motivation to exercise compared to athletes that experienced no change in motivation. State happiness describes the hedonic perspective of making progress toward a goal that we are striving for and can lead to goal-oriented behavior (Lazarus, 2000). Also, positive affect, with respect to PSI theory, employs less self-regulatory capacities, makes it easier to access the extension memory system including stored action plans, and finally, requires less self-control to access the behavioral control relevant for executing routines (Kuhl, 2018). Therefore, actually exercising or being motivated to exercise should be less impacted. Additionally, it could help explain why the level of relaxation is also significantly lower.

Interestingly, athletes more motivated to exercise are descriptively the happiest and the most relaxed, which could be considered contradictory to our previous speculation that they might use sport as some sort of compensation. However, it is important to keep in mind that compensation is not necessarily negative. Exercise is known to have rapid positive impact on mood (see meta analyses Jansen and Hoja, 2018). Thus, the increased motivation to exercise might actually lead to more exercise and thus, a positive impact on mood. This is highly speculative in nature, since we did not assess their actual exercise frequency.

### The Interaction of State and Trait Psychological Variables

Combining findings on state and trait variables a picture emerges that is in line with theories on the impact of stress/emotions on motivation, stress and personality (e.g., PSI theory; approach-avoidance temperament). For example, focusing on personality, previous studies have shown that neuroticism is related to distress, more negative emotions and emotion-focused coping whereas conscientiousness predicts task-oriented coping (Matthews et al., 2006). In addition, individuals with higher neuroticism have more difficulties handling their distress (O’Brien and DeLongis, 1996). Finally, higher levels of neuroticism are associated with avoidance motivation (Elliot and Thrash, 2010). The same pattern can be detected in our data.

Neuroticism is significantly positively correlated with trait anxiety (*r* = 0.559, *p* < 0.001), state anxiety (*r* = 0.271, *p* = 0.008) and perceived stress (*r* = 0.392, *p* < 0.001) as well as negatively correlated with self-efficacy to handle stress (*r* = −0.302, *p* = 0.003). Also, negative emotions and feelings are often related to avoidance motivation (see Elliot et al., 2013). Thus unsurprisingly, amateur and recreational athletes with the highest negative emotions were less motivated to train. Finally, extraversion is typically linked to approach motivation (Elliot and Thrash, 2010) and is related to interpersonal coping strategies (Matthews et al., 2006). This fits with our data as we also found that extraversion is significantly related to distraction-social-oriented coping (*p* < 0.001, *r* = 0.524). Overall, athletes that are the least emotionally affected by the pandemic and the lockdown show a different profile of psychological trait variables. It can be speculated that might be the reason for their stable motivation throughout this time of crisis.

### Problems, Coping Strategies, Wanted and Received External Support and Joyful Anticipation

Overall, closed sport facilities, limited social contacts and difficulties maintaining physical shape were the most often mentioned problems, and these are in line with those anticipated by experts (e.g., Mehrsafar et al., 2020; Schinke et al., 2020; Spitzer, 2020). Athletes coped with these difficulties by exercising online, organizing and structuring their day and using online tools to stay in contact with others. This is also in line with coping strategies suggested by experts (e.g., Jukic et al., 2020). Most athletes received support from family, friends, and significant others which can be summarized as emotional support and is highly typical for crisis situations (Shumaker and Brownell, 1984). Athletes wished for more help from their coaches, the state, and universities which can be summarized as missing informational and instrumental support (Shumaker and Brownell, 1984). Finally, athletes were most looking forward to social contacts, leisure activities, and practicing sport with others. These findings show that humans are social creatures and highly enjoy the pleasures of life. In turn, missing these activities could lead to their higher appreciation and to a further strengthening of relationships (e.g., coach-athlete; Li et al., 2020).

Descriptive differences between groups were detected concerning problems, coping strategies, and joyful anticipations. First, athletes who reported less motivation to exercise were aware of this issue as almost 50% also explicitly stated that lack of exercise was as a problem. In addition, they stated less that they missed social contacts and were less concerned about closed sport facilities. At first glance, this might be cause for concern as these are also symptoms of depression. However, 88% of the members of this group also stated that they were joyfully anticipating social contacts, which suggests that we do not yet have a reason to be concerned. However, the uncertainty of a second lockdown, other restrictions, or further social isolation could lead to mental health issues. Secondly, athletes who reported less motivation did not mention the use of online training tools as often. This is not surprising and may be indicative of that fact that less motivated athletes also actually exercise less.

### Limitation

This study has several limitations and has been conducted under immense time pressure (see also Brand et al., 2020). As a cross-sectional study it allows for no causal conclusions, strictly speaking. Moreover, all data are self-reported and this can lead to bias. Additionally, data were assessed online and this led to selection bias in the sampling process (Bethlehem, 2010). In the same vein, our sample consisted of young athletes on average 22 years of age, which is rather small compared to recent studies on the topic. Thus, results should not be translated to younger or older individuals. However, effect sizes for detected differences are medium to large, which can be considered rather positive in terms of relevance of our results. Nevertheless, our main variable –motivation to exercise compared to pre-COVID-19 – was assessed by a single item only. Nevertheless, with reference to Brand et al. (2020), we were also more interested in generally perceived changes in exercise motivation and in retrospectively questioning the finding that the amount of training leads to biases, poor memory recall, and potential exaggerations. Lastly, only a preselected number of trait variables have been analyzed and the BFI-10 (Rammstedt et al., 2013) shows partially unacceptable Cronbach α-values. Thus, our personality trait results should be interpreted very carefully, even though, this inventory has already contributed to other studies as a useful short-questionnaire (e.g., Mauz et al., 2017). Finally, in future research, listed trait variables could be expanded for example, to include emotional intelligence or resilience, as these personality traits have been shown to have a positive effect on maintaining physical activity during the COVID-19 pandemic (Mon-López et al., 2020).

## Conclusion

Our study investigated stress, coping, and changes in motivation to exercise in amateur and recreational athletes during the first COVID-19 lockdown (March–April 2020) in Germany. We found that amateur and recreational athletes experienced an increase in stress due to closed sporting facilities and limited social contacts. They experiencing the least stress due to the lockdown, however, showed not only no change in motivation but also presented a different, potentially more positive profile in trait variables which allows us to speculate that this might be the reason for the stability in motivation (in accordance with Funke, 2020). Nevertheless, athletes’ self-efficacy was maintained and they reported several coping strategies, mainly using online tools for exercise and for keeping socially connected. Even though our study provides data on the relationship between stress, coping, personality, and motivation, we are convinced that we still need theoretical models relevant for such a crisis and for this specific group (i.e., amateur and recreational athletes). Furthermore, we urgently need intervention measures to support our amateur and recreational athletes so they can master such a crisis of uncertainty. Applied sport psychologists consider this a chance to shift away from deficit-oriented to resource-oriented sport psychological practices that focus more on athletes’ well-being (see AASP (Association for Applied Sport Psychology) Blog, 2020). Especially, Acceptance and Commitment Therapy (AASP (Association for Applied Sport Psychology) Blog, 2020) as well as salutogenic approaches (Leisterer et al., under review) might be beneficial in view of further emerging crises. Given the limitations of our study, we tentatively suggest that based on our results, new approaches ought to focus on supporting athletes’ staff (especially coaches) to foster athletes’ management of daily obstacles (e.g., closed sport facilities). In addition, athletes as well as their staff should learn how to elicit resilience and motivation. Further, coaches and staff should keep an eye on conspicuously more motivated athletes during the COVID-19 pandemic. Overall, athletes as well as their staff should be in the focus of further research, for example in devising targeted workshops (e.g., Leisterer et al., under review) and guidelines (e.g., Bertollo et al., 2020), which attempt to overcome negative impacts of the COVID-19 pandemic.

## Data Availability Statement

The raw data supporting the conclusions of this article will be made available by the authors, without undue reservation.

## Ethics Statement

Ethical review and approval was not required for the study on human participants in accordance with the local legislation and institutional requirements. The patients/participants provided their written informed consent to participate in this study.

## Author Contributions

FL and SL: idea and conceptualization. FL: planning of the study and first draft. SL, NW, A-ME, and OL: revisions. FL and SG: literature research. FL, VP, and OL: data analysis. LK and TM: formatting. A-ME: proofreading. A-ME and FL: resources and supervision. All authors contributed to the article and approved the submitted version.

## Conflict of Interest

The authors declare that the research was conducted in the absence of any commercial or financial relationships that could be construed as a potential conflict of interest.

## Footnotes

1 The lack of motivation is related to class work.

## References

[B1] AASP (Association for Applied Sport Psychology) Blog (2020). *How Olympic and Paralympic Athletes are Adjusting With the Help of Sport Psychology During the Coronavirus Outbreak.* Available online at: https://appliedsportpsych.org/blog/2020/05/olympic-and-paralympic-athletes-are-adjusting-with-the-help-of-sport-psychology-during-the-coronavirus-outbreak/ (accessed September 20, 2020).

[B2] Andreato L. V. Coimbra D. R. Andrade A. (2020). Challenges to athletes during the home confinement caused by the COVID-19 pandemic. *Strength Cond. J.* 42 1–5. 10.1519/SSC.0000000000000563

[B3] Angosto S. Berengüí R. Vegara-Ferri J. M. López-Gullón J. M. (2020). Motives and commitment to sport in amateurs during confinement: a segmentation study. *Int. J. Environ. Res. Public Health* 17:20. 10.3390/ijerph17207398 33050616 PMC7600813

[B4] Batey J. Parry K. (2020). *Coronavirus: Why Self-Isolation Brings Mental Health Strain for Elite Athletes.* Available online at: https://theconversation.com/coronavirus-why-self-isolation-brings-mental-health-strain-for-elite-athletes-135273 (accessed September 20, 2020).

[B5] Bertollo M. Forzini F. Conti C. Bondár R. Z. (2020). *Behavioural Indication for Cyclists During COVID-19 Lockdown.* Available online at: https://www.researchgate.net/publication/341266159_Behavioural_indication_for_cyclists_during_COVID-19_lockdown (accessed September 20, 2020)

[B6] Bethlehem J. (2010). Selection bias in web surveys. *Int. Stat. Rev.* 78 161–188. 10.1111/j.1751-5823.2010.00112.x

[B7] Bortz J. Döring N. (2006). *Forschungsmethoden und Evaluation: Für Human- und Sozialwissenschaftler [Research Methods and Evaluation: For Human and Social Scientists].* Heidelberg: Springer-Verlag.

[B8] Bowes A. Lomax L. Piasecki J. (2020). The impact of the COVID-19 lockdown on elite sportswomen. *Manag. Sport Leis.* 1–17. 10.1080/23750472.2020.1825988

[B9] Brand R. Timme S. Nosrat S. (2020). When pandemic hits: exercise frequency and subjective well-being during COVID-19 pandemic. *Front. Psychol.* 11:570567. 10.3389/fpsyg.2020.570567 33071902 PMC7541696

[B10] Braun V. Clarke V. Weate P. (2016). “Using thematic analysis in sport and exercise research,” in *Routledge Handbook of Qualitative Research in Sport and Exercise*, eds Smith B. Sparkes A. C. (Abingdon: Routledge), 191–205.

[B11] Brewer B. W. Van Raalte J. L. Linder D. E. (1993). Athletic identity: Hercules’ muscles or achilles heel? *Int. J. Sport Psychol.* 24 237–254.

[B12] Clemente-Suárez V. J. Fuentes-García J. P. de la Vega M. R. Martínez P. (2020). Modulators of the personal and professional threat perception of Olympic athletes in the actual COVID-19 crisis. *Front. Psychol.* 11:1985. 10.3389/fpsyg.2020.01985 32849157 PMC7419607

[B13] Costa S. Santi G. di Fronso S. Montesano C. di Gruttola F. Ciofi E. G. (2020). Athletes and adversities: athletic identity and emotional regulation in time of COVID-19. *Sport Sci. Health* 31 609–618. 10.1007/s11332-020-00677-9 32904823 PMC7457888

[B14] Creswell J. W. Hanson W. E. Clark Plano V. L. Morales A. (2007). Qualitative research designs: selection and implementation. *Couns. Psychol.* 35 236–264. 10.1177/0011000006287390

[B15] Deci E. Ryan R. (2000). The “what” and “why” of goal pursuits: human needs and the self-determination of behavior. *Psychol. Inq.* 11 227–268. 10.1207/s15327965pli1104_01

[B16] Di Fronso S. Costa S. Montesano C. Di Gruttola F. Giorgio Ciofi E. Morgilli L. (2020). The effects of COVID-19 pandemic on perceived stress and psychobiosocial states in Italian athletes. *Int. J. Sport Exerc. Psychol.* 1–13. 10.1080/1612197X.2020.1802612

[B17] Elbe A.-M. Wenhold F. (2005). Cross-cultural test control criteria for the AMS-Sport. *Int. J. Sport Exerc. Psychol.* 3 163–177. 10.1080/1612197x.2005.9671765

[B18] Elliot A. J. Thrash T. M. (2010). Approach and avoidance temperament as basic dimensions of personality. *J. Pers.* 78 865–906. 10.1111/j.1467-6494.2010.00636.x 20573129

[B19] Elliot A. J. Eder A. B. Harmon-Jones E. (2013). Approach–avoidance motivation and emotion: convergence and divergence. *Emot. Rev.* 5 308–311. 10.1177/1754073913477517

[B20] Feltz D. L. (2007). “Self-confidence and sports performance,” in *Essential Readings in Sport and Exercise Psychology*, eds Smith D. Bar-Eli M. (Champaign, IL: Human Kinetics), 278–294.

[B21] Filipas L. La Torre A. Luzi L. Codella R. (2020). Born to run out of COVID-19: what gives us wings. *Front. Sports Act. Living* 2:105. 10.3389/fspor.2020.00105 33345094 PMC7739669

[B22] Frank A. Hörmann S. Krombach J. Fatke B. Holzhüter F. Frank W. (2020). Psychisch krank in Krisenzeiten: Subjektive Belastungen durch COVID-19. [Psychologically ill in times of crises: Subjective stress during COVID-19.] *Psychiatr. Prax.* 47 267–272. 10.1055/a-1179-4230 32542637

[B23] Funke J. (2020). Entwicklung einer pandemie: psychologische aspekte der corona-krise [Development of a pandemic: psychological aspects during corona crises]. *Heidelberger Jahrbücher Online* 5 219–246. 10.17885/heiup.hdjbo.2020.0.24181

[B24] Gerber M. Isoard-Gautheur S. Schilling R. Ludyga S. Brand S. Colledge F. (2018). When low leisure-time physical activity meets unsatisfied psychological needs: insights from a stress-buffer perspective. *Front. Psychol.* 9:2097. 10.3389/fpsyg.2018.02097 30450065 PMC6224427

[B25] González-Valero G. Zurita-Ortega F. Lindell-Postigo D. Conde-Pipó J. Grosz W. R. Badicu G. (2020). Analysis of self-concept in adolescents before and during COVID-19 lockdown: differences by gender and sports activity. *Sustainability* 12:7792 10.3390/su12187792

[B26] Grimm J. (2009). *State-Trait-Anxiety Inventory Nach Spielberger: Deutsche Lang- und Kurzversion. [Long and Short Version in German]. MF-Working Paper.* Vienna: Methodenforum der Universität Wien.

[B27] Håkansson A. Jönsson C. Kenttä G. (2020). Psychological distress and problem gambling in elite athletes during COVID-19 restrictions—a web survey in top leagues of three sports during the pandemic. *Int. J. Environ. Res. Public Health.* 17:6693. 10.3390/ijerph17186693 32937978 PMC7559357

[B28] Harmon-Jones C. Bastian B. Harmon-Jones E. (2016). The discrete emotions questionnaire: a new tool for measuring state self-reported emotions. *PLoS One.* 11:e0159915. 10.1371/journal.pone.0159915 27500829 PMC4976910

[B29] Heckhausen J. Heckhausen H. (2008). *Motivation and Action.* Heidelberg: Springer-Verlag, 10.1017/CBO9780511499821

[B30] Henriksen K. Schinke R. J. Moesch K. McCann S. Parham W. D. Larsen C. H. (2019). Consensus statement on improving mental health of high performance athletes. *Int. J. Sport Exerc. Psychol.* 18 553–560. 10.1080/1612197X.2019.1570473

[B31] Henriksen K. Schinke R. McCann S. Durand-Bush N. Moesch K. Parham W. D. (2020). Athlete mental health in the Olympic/Paralympic quadrennium: a multi-societal consensus statement. *Int. J. Sport Exerc. Psychol.* 18 391–408. 10.1080/1612197X.2020.1746379

[B32] Iancheva T. Rogaleva L. Garcia-Mas A. Olmedilla A. (2020). Perfectionism, mood states, and coping strategies of sports students from Bulgaria and Russia during the pandemic Covid-19. *J. Appl. Sport Sci.* 1 22–38. 10.37393/JASS.2020.01.2

[B33] Jansen P. Hoja S. (2018). Macht Sport wirklich glücklich? Ein systematisches Review [Does exercise really make us happy? A systematic review]. *Zeitschrift Sportpsychol.* 25 21–32. 10.1026/1612-5010/a000211

[B34] Jukic I. Calleja-González J. Cos F. Cuzzolin F. Olmo J. Terrados N. (2020). Strategies and solutions for team sports athletes in isolation due to COVID-19. *Sports* 8:56. 10.3390/sports8040056 32344657 PMC7240607

[B35] Kälin W. (1995). *in Deutsche 24-ltem Kurzform des Coping Inventory for Stressful Situations” (CISS)“ [German 24 Item Short Version of the Coping Inventory for Stressful Situations]*, eds Endler N. S. Parker J. D. A. (Bern: University of Bern Institute of Psychology). transl Semmer N. Tschan F. Schade V. 10.3390/sports8040056

[B36] Kazén M. Quirin M. (2018). “The integration of motivation and volition in personality systems interactions (PSI) theory,” in *Why People Do the Things They Do: Building on Julius Kuhl’s Contributions to the Psychology of Motivation and Volition*, eds Baumann N. Kazeìn M. Quirin M. Koole S. L. (Göttingen: Hogrefe), 15–30.

[B37] Klein E. M. Brähler E. Dreier M. Reinecke L. Müller K. W. Schmutzer G. (2016). The German version of the perceived stress scale – psychometric characteristics in a representative German community sample. *BMC Psychiatry* 16:159. 10.1186/s12888-016-0875-9 27216151 PMC4877813

[B38] Kuhl J. (2018). “Individuelle unterschiede in der selbststeuerung [Individual differences in self-control],” in *Motivation and Action*, eds Heckhausen J. Heckhausen H. (Heidelberg: Springer-Verlag), 389–422. 10.1007/978-3-662-53927-9_13

[B39] Laborde S. Guillén F. Mosley E. (2016). Positive personality-trait-like individual differences in athletes from individual-and team sports and in non-athletes. *Psychol. Sport Exerc.* 26 9–13. 10.1016/j.psychsport.2016.05.009

[B40] Lautenbach F. Laborde S. Putman P. Angelidis A. Raab M. (2016). Attentional distraction from negative sports words in athletes under low- and high-pressure conditions: evidence from the sport emotional stroop task. *Int. J. Sport Exerc. Psychol.* 5 296–307. 10.1037/spy0000073

[B41] Lazarus R. S. (2000). How emotions influence performance in competitive sports. *Sport Psychol.* 14 229–252. 10.1123/tsp.14.3.229

[B42] Leite E. C. P. (2018). *The Relationship Between Leadership, Orientation to Happiness and Work Motivation.* Ph.D. dissertation, University of Coimbra, Coimbra.

[B43] Lewin K. (1935). *A Dynamic Theory of Personality.* New York, NY: McGraw-Hill.

[B44] Li J. Gao H. Liu P. Zhong C. (2020). Does distance produce beauty? The influence of COVID-19 lockdown on the coach-athlete relationship in a Chinese football school. *Front. Psychol.* 11:560638. 10.3389/fpsyg.2020.560638 33013600 PMC7516042

[B45] Lim M. A. Pranata R. (2020). Sports activities during any pandemic lockdown. *Ir. J. Med. Sci.* 4 1–5. 10.1007/s11845-020-02300-9 32621168 PMC7334119

[B46] Maher J. P. Hevel D. J. Reifsteck E. J. Drollette E. S. (2020). Physical activity is positively associated with college students‘ positive affect regardless of stressful life events during the COVID-19 pandemic. *Psychol. Sport Exerc.* 52:101826. 10.1016/j.psychsport.2020.101826 33100905 PMC7568511

[B47] Makarowski R. Piotrowski A. Predoiu R. Görner K. Predoiu A. Mitrache G. (2020). Stress and coping during the COVID-19 pandemic among martial arts athletes–a cross-cultural study. *Arch. Budo.* 16 161–171.

[B48] Mascret N. (2020). Confinement during Covid-19 outbreak modifies athletes’ self-based goals. *Psychol. Sport Exerc.* 51:101796. 10.1016/j.psychsport.2020.101796 32922209 PMC7475734

[B49] Matthews G. Emo A. K. Funke G. Zeidner M. Roberts R. D. Costa P. T. Jr. (2006). Emotional intelligence, personality, and task-induced stress. *J. Exp. Psychol.* 12 96–107. 10.1037/1076-898X.12.2.96 16802891

[B50] Maugeri G. Castrogiovanni P. Battaglia G. Pippi R. D’Agata V. Palma A. (2020). The impact of physical activity on psychological health during COVID-19 pandemic in Italy. *Heliyon* 6:e04315. 10.1016/j.heliyon.2020.e04315 32613133 PMC7311901

[B51] Mauz E. Gößwald A. Kamtsiuris P. Hoffmann R. Lange M. Schenck U. v. (2017). New data for action. Data collection for KiGGS Wave 2 has been completed. *J. Health Monitor.* 2 2–27. 10.17886/RKI-GBE-2017-105 PMC10291840 37377941

[B52] Mehrsafar A. H. Gazerani P. Zadeh A. M. Sanchez J. C. J. (2020). Addressing potential impact of COVID-19 pandemic on physical and mental health of elite athletes. *Brain Behav. Immun.* 87 147–148. 10.1016/j.bbi.2020.05.011 32387513 PMC7201218

[B53] Mon-López D. de la Rubia Riaza A. Hontoria Galán M. Refoyo Roman I. (2020). The impact of COVID-19 and the effect of psychological factors on training conditions of handball players. *Int. J. Environ. Res. Public Health.* 17:6471. 10.3390/ijerph17186471 32899526 PMC7558666

[B54] Mutz M. (2020). Forced adaptations of sporting behaviours during the COVID-19 pandemic and their effects on subjective well-being. *Eur. Soc.* 1–15. 10.1080/14616696.2020.1821077

[B55] Mutz M. Gerke M. (2020). Sport and exercise in times of self-quarantine: How Germans changed their behavior at the beginning of the Covid-19 pandemic. *Int. Rev. Sport Sociol.* 1012690220934335 10.1177/1012690220934335

[B56] O’Brien T. B. DeLongis A. (1996). The interactional context of problem-, emotion-, and relationship-focused coping: the role of the Big Five personality factors. *J. Pers.* 64 775–813. 10.1111/j.1467-6494.1996.tb00944.x 8956513

[B57] Paoli A. Musumeci G. (2020). Elite athletes and COVID-19 lockdown: future health concerns for an entire sector. *J. Funct. Morphol. Kinesiol.* 5:30 10.3390/jfmk5020030 PMC7739347 33467246

[B58] Peterson C. Ruch W. Beermann U. Park N. Seigman M. E. P. (2007). Strength of character, orientations to happiness, and life satisfaction. *J. Posit. Psychol.* 2 149–156. 10.1080/17439760701228938

[B59] Pinto A. J. Dunstan D. W. Owen N. Bonfa E. Gualano B. (2020). Combating physical inactivity during the COVID-19 pandemic. *Rheumatology* 16 347–348. 10.1038/s41584-020-0427-z 32355296 PMC7191971

[B60] Pivarnik J. M. Reeves M. J. Rafferty A. P. (2003). Seasonal variation in adult leisure-time physical activity. *Med. Sci. Sports Exerc.* 35 1004–1008. 10.1249/01.mss.0000069747.55950.b1 12783049

[B61] Rammstedt B. Kemper C. J. Klein C. Beierlein C. Kovaleva A. (2013). Eine kurze skala zur messung der fünf dimensionen der persönlichkeit: 10 item big five inventory (BFI-10) [A short scale for assessing the big five dimensions of personality: 10 items big five inventory (BFI-10)]. *Method Daten Anal.* 7 233–249. 10.12758/mda.2013.013

[B62] Rea S. (2020). *Coronavirus: How Can Athletes get Through This Period of Isolation?.* Available online at: https://www.open.edu/openlearn/health-sports-psychology/coronavirus-how-can-athletes-get-through-period-isolation (accessed September 20, 2020).

[B63] Ruch W. Martínez-Marti M. L. Heintz S. Brouwers S. A. (2014). Short form of the orientations to happiness questionnaire for the German-speaking countries: development and analysis of the psychometric properties. *Swiss J. Psychol.* 73 225–234. 10.1024/1421-0185/a000xxx

[B64] Samuel R. D. Tenenbaum G. Galily Y. (2020). The 2020 Coronavirus pandemic as a change-event in sport performers’ careers: conceptual and applied practice considerations. *Front. Psychol.* 11:567966. 10.3389/fpsyg.2020.567966 33071895 PMC7540073

[B65] Scarantion A. (2018). “The philosophy of emotions and its impact on affective science,” in *Handbook of Emotions*, eds Feldman Barrett L. Lewis M. Haviland-Jones J. M. (New York, NY: The Guilford Press), 3–48.

[B66] Schinke R. Papaioannou A. Maher C. Parham W. D. Larsen C. H. Gordin R. (2020). Sport psychology services to professional athletes: working through COVID-19. *Int. J. Sport Exerc. Psychol.* 18 409–413. 10.1080/1612197X.2020.1766182

[B67] Schmid J. Seiler R. (2003). Identität im hochleistungssport: überprüfung einer deutschsprachigen adaptation der athletic identity measurement scale (AIMS-D) [identity in high-performance sport: psychometric investigations with a German language adaptation of the athletic identity measurement scale (AIMS-D)]. *Diagnostica* 49 176–183. 10.1026//0012-1924.49.4.176

[B68] Şenışık S. Denerel N. Köyağasıoğlu O. Tunç S. (2020). The effect of isolation on athletes’ mental health during the COVID-19 pandemic. *Phys. Sportsmed.* 10.1080/00913847.2020.1807297 32762510

[B69] Shumaker S. A. Brownell A. (1984). Toward a theory of social support: closing conceptual gaps. *J. Soc. Issues* 40 11–36. 10.1111/j.1540-4560.1984.tb01105.x

[B70] Slimani M. Paravlic A. Mbarek F. Bragazzi N. L. Tod D. (2020). The relationship between physical activity and quality of life during the confinement induced by COVID-19 outbreak: a pilot study in Tunisia. *Front. Psychol.* 11:1882. 10.3389/fpsyg.2020.01882 32849104 PMC7427614

[B71] Spitzer M. (2020). Psychologie und pandemie. die auswirkungen des Corona-Virus auf den einzelnen und auf die gesellschaft [Psychology and pandemic. The impact of the corona virus on the individual and sociciety]. *Nervenheilkunde* 39 274–283. 10.1055/a-1094-9461

[B72] Stambulova N. (2011). The mobilization model of counseling athletes in crisis-transitions: an educational intervention tool. *J. Sport Psychol. Action* 2 156–170. 10.1080/21520704.2011.598223

[B73] Stambulova N. B. Schinke R. J. Lavallee D. Wylleman P. (2020). The COVID-19 pandemic and Olympic/Paralympic athletes‘development challenges and possibilities in times of global crisis-transition. *Int. J. Sport Exerc. Psychol.* 1–10. 10.1080/1612197X.2020.1810865

[B74] Tingaz E. O. (2020). The psychological impact of COVID-19 pandemic on elite athletes, management strategies and post-pandemic performance expectations: a semi structured interview study. *Int. J. Educ. Res. Inn.* 15 73–81. 10.46661/ijeri.4863

[B75] Tittles C. R. (1995). *Control Balance: Toward a General Theory of Deviance.* Boulder, Co: Westview.

[B76] Weiner B. (1985). An attributional theory of achievement motivation and emotion. *Psych. Rev.* 92 548–573. 10.1037/0033-295x.92.4.548 3903815

[B77] Wylleman P. (2020). *Wie Bleibe ich Positiv [How to stay positive].* Available online at: https://www.olympic.org/athlete365/de/well-being-de/how-to-stay-positive/ (accessed September 20, 2020)

[B78] Yang J. Tong J. Meng F. Feng Q. Ma H. Shi C. (2020). Characteristics and challenges of psychological first aid in China during the COVID-19 outbreak. *Brain Behav. Immun.* 87 113–114. 10.1016/j.bbi.2020.04.075 32353519 PMC7185020

[B79] Yang Y. Li P. Fu X. Kou Y. (2017). Orientations to happiness and subjective well-being in Chinese adolescents: The roles of prosocial behavior and internet addictive behavior. *J. Happiness Stud.* 18 1747–1762. 10.1007/s10902-016-9794-1

