# Supplementary material for: Amateur and Recreational Athletes’ Motivation to Exercise, Stress, and Coping During the Corona Crisis
Source: Front Psychol. 2021 Jan 27;11:611658. doi: 10.3389/fpsyg.2020.611658 (PMC7873522; doi:10.3389/fpsyg.2020.611658)
Supplement: Supplementary file 1 [file Table_1.DOCX]

Supplementary Material

Table 1

*Frequencies of problems (i.e., perceived challenges) for the complete sample and depending on motivational changes due to the lockdown*

| Problems | Change in motivation to exercise | *n* | *%* |
| --- | --- | --- | --- |
| Sports facilities closed | Less (*n*=96) | **15** | **45.45^2^** |
|  | Same (*n*=111) | **30** | **76.921^1^** |
|  | More (*n*=63) | **16** | **69.60^1^** |
|  | Total sample (*N*=270) | **61** | **64.21^1^** |
| Social contacts | Less (*n*=96) | 8 | 24.24 |
|  | Same (*n*=111) | **19** | **48.72^2^** |
|  | More (*n*=63) | **8** | **34.78^2^** |
|  | Total sample (*N*=270) | **35** | **36.84^2^** |
| Staying fit | Less (*n*=96) | **9** | **27.27^3^** |
|  | Same (*n*=111) | **12** | **30.77^3^** |
|  | More (*n*=63) | 4 | 17.39 |
|  | Total sample (*N*=270) | **25** | **26.32^3^** |
| Motivation | Less (*n*=96) | **16** | **48.48^1^** |
|  | Same (*n*=111) | 5 | 12.82 |
|  | More (*n*=63) | 2 | 8.70 |
|  | Total sample (*N*=270) | 23 | 24.21 |
| Team | Less (*n*=96) | 6 | 18.18 |
|  | Same (*n*=111) | 5 | 12.82 |
|  | More (*n*=63) | **8** | **34.78^3^** |
|  | Total sample (*N*=270) | 19 | 20.00 |
| Train alone | Less (*n*=96) | 5 | 15.15 |
|  | Same (*n*=111) | 5 | 12.82 |
|  | More (*n*=63) | 5 | 21.74 |
|  | Total sample (*N*=270) | 15 | 15.79 |
| Organization | Less (*n*=96) | 7 | 21.21 |
|  | Same (*n*=111) | 3 | 7.69 |
|  | More (*n*=63) | 3 | 13.04 |
|  | Total sample (*N*=270) | 13 | 13.68 |
| Equipment | Less (*n*=96) | 7 | 21.21 |
|  | Same (*n*=111) | 3 | 7.69 |
|  | More (*n*=63) | 2 | 8.70 |
|  | Total sample (*N*=270) | 12 | 12.63 |
| Education | Less (*n*=96) | 5 | 15.15 |
|  | Same (*n*=111) | 5 | 12.82 |
|  | More (*n*=63) | 1 | 4.35 |
|  | Total sample (*N*=270) | 11 | 11.58 |
| Uncertainty | Less (*n*=96) | 5 | 15.15 |
|  | Same (*n*=111) | 3 | 7.69 |
|  | More (*n*=63) | 2 | 8.70 |
|  | Total sample (*N*=270) | 10 | 10.53 |
| Everyday Life | Less (*n*=96) | 5 | 15.15 |
|  | Same (*n*=111) | 2 | 5.13 |
|  | More (*n*=63) | 3 | 13.04 |
|  | Total sample (*N*=270) | 10 | 10.53 |
| Missing Competitions | Less (*n*=96) | 2 | 6.06 |
|  | Same (*n*=111) | 6 | 15.38 |
|  | More (*n*=63) | 1 | 4.35 |
|  | Total sample (*N*=270) | 9 | 9.47 |
| Variety | Less (*n*=96) | 0 | 0 |
|  | Same (*n*=111) | 6 | 15.38 |
|  | More (*n*=63) | 3 | 13.04 |
|  | Total sample (*N*=270) | 9 | 9.47 |
| Financial worries | Less (*n*=96) | 3 | 9.09 |
|  | Same (*n*=111) | 1 | 2.56 |
|  | More (*n*=63) | 4 | 17.39 |
|  | Total sample (*N*=270) | 8 | 8.42 |
| No Coach | Less (*n*=96) | 3 | 9.09 |
|  | Same (*n*=111) | 2 | 5.13 |
|  | More (*n*=63) | 1 | 4.35 |
|  | Total sample (*N*=270) | 6 | 6.32 |
| Nutrition | Less (*n*=96) | 0 | 0 |
|  | Same (*n*=111) | 2 | 5.13 |
|  | More (*n*=63) | 0 | 0 |
|  | Total sample (*N*=270) | 2 | 2.11 |
| No perceived Challenges | Less (*n*=96) | 0 | 0 |
|  | Same (*n*=111) | 2 | 5.12 |
|  | More (*n*=63) | 0 | 0 |
|  | Total sample (*N*=270) | 2 | 2.11 |

*Note*. ^1^ = challenge with the highest frequency for this subgroup; ^2^ = Challenge with the second highest frequency; ^3^ = Challenge with the third-highest frequency

Table 2

*Frequencies of coping strategies for the complete sample and depending on motivational changes due to the lockdown*

| Coping strategy | Change in motivation to exercise | *n* | *%* |
| --- | --- | --- | --- |
| Online sport courses | Less (*n*=91) | **11** | **33.33^2^** |
|  | Same (*n*=110) | **30** | **76.92^1^** |
|  | More (*n*=67) | **22** | **95.65^1^** |
|  | Total sample (*N*=268) | **63** | **96.84^1^** |
| Organization | Less (*n*=91) | **14** | **42.42^1^** |
|  | Same (*n*=110) | **14** | **35.90^2^** |
|  | More (*n*=67) | **16** | **69.57^2^** |
|  | Total sample (*N*=268) | **44** | **61.05^2^** |
| Social contacts | Less (*n*=91) | **10** | **30.30^3^** |
|  | Same (*n*=110) | **13** | **33.33^3^** |
|  | More (*n*=67) | **6** | **26.09^3^** |
|  | Total sample (*N*=268) | **29** | **44.21^3^** |
| Alternative sports | Less (*n*=91) | 5 | 15.15 |
|  | Same (*n*=110) | 11 | 28.21 |
|  | More (*n*=67) | 4 | 17.39 |
|  | Total sample (*N*=268) | 20 | 32.63 |
| Positive thinking | Less (*n*=91) | 7 | 21.21 |
|  | Same (*n*=110) | 8 | 20.51 |
|  | More (*n*=67) | 0 | 0 |
|  | Total sample (*N*=268) | 15 | 24.21 |
| Goal setting | Less (*n*=91) | 7 | 21.21 |
|  | Same (*n*=110) | 3 | 7.69 |
|  | More (*n*=67) | 1 | 4.35 |
|  | Total sample (*N*=268) | 11 | 15.79 |
| Discipline | Less (*n*=91) | 5 | 15.15 |
|  | Same (*n*=110) | 2 | 5.13 |
|  | More (*n*=67) | 4 | 17.39 |
|  | Total sample (*N*=268) | 11 | 14.74 |
| Hobby/Distraction | Less (*n*=91) | 5 | 15.15 |
|  | Same (*n*=110) | 2 | 5.13 |
|  | More (*n*=67) | 3 | 13.04 |
|  | Total sample (*N*=268) | 10 | 13.68 |
| Train with Others | Less (*n*=91) | 3 | 9.09 |
|  | Same (*n*=110) | 5 | 12.82 |
|  | More (*n*=67) | 2 | 8.70 |
|  | Total sample (*N*=268) | 10 | 13.68 |
| Be outside | Less (*n*=91) | 5 | 15.15 |
|  | Same (*n*=110) | 3 | 7.69 |
|  | More (*n*=67) | 1 | 4.35 |
|  | Total sample (*N*=268) | 9 | 12.63 |
| Acceptance | Less (*n*=91) | 3 | 9.09 |
|  | Same (*n*=110) | 3 | 7.69 |
|  | More (*n*=67) | 2 | 8.70 |
|  | Total sample (*N*=268) | 8 | 11.58 |
| Nutrition | Less (*n*=91) | 1 | 3.03 |
|  | Same (*n*=110) | 4 | 10.26 |
|  | More (*n*=67) | 3 | 13.04 |
|  | Total sample (*N*=268) | 8 | 11.58 |
| Avoidance | Less (*n*=91) | 5 | 15.15 |
|  | Same (*n*=110) | 2 | 5.13 |
|  | More (*n*=67) | 0 | 0 |
|  | Total sample (*N*=268) | 7 | 10.53 |
| Mental | Less (*n*=91) | 4 | 12.12 |
|  | Same (*n*=110) | 3 | 7.69 |
|  | More (*n*=67) | 0 | 0 |
|  | Total sample (*N*=268) | 7 | 9.47 |
| Music/Movies | Less (*n*=91) | 1 | 3.03 |
|  | Same (*n*=110) | 3 | 7.69 |
|  | More (*n*=67) | 1 | 4.35 |
|  | Total sample (*N*=268) | 5 | 8.42 |
| Further education | Less (*n*=91) | 1 | 3.03 |
|  | Same (*n*=110) | 2 | 5.13 |
|  | More (*n*=67) | 0 | 0 |
|  | Total sample (*N*=268) | 3 | 5.26 |
| Hope | Less (*n*=91) | 3 | 9.09 |
|  | Same (*n*=110) | 0 | 0 |
|  | More (*n*=67) | 0 | 0 |
|  | Total sample (*N*=268) | 3 | 4.21 |
| Patience | Less (*n*=91) | 1 | 3.03 |
|  | Same (*n*=110) | 1 | 2.56 |
|  | More (*n*=67) | 1 | 4.35 |
|  | Total sample (*N*=268) | 3 | 3.16 |
| Reading | Less (*n*=91) | 0 | 0 |
|  | Same (*n*=110) | 1 | 2.56 |
|  | More (*n*=67) | 1 | 4.35 |
|  | Total sample (*N*=268) | 2 | 3.16 |

*Note*. ^1^ = coping strategies with the highest frequency for this subgroup; ^2^ = coping strategies with the second highest frequency; ^3^ = coping strategies with the third-highest frequency

Table 3

*Frequencies of received help for the complete sample and depending on motivational changes due to the lockdown*

| Received help from… | Change in motivation to exercise | *n* | *%* |
| --- | --- | --- | --- |
| Family | Less (*n*=45) | **13** | **39.39^1^** |
|  | Same (*n*=51) | **20** | **51.28^1^** |
|  | More (*n*=26) | **7** | **30.43^1^** |
|  | Total sample (*N*=122) | **40** | **42.11^1^** |
| Friends | Less (*n*=45) | **10** | **30.30^2^** |
|  | Same (*n*=51) | **7** | **17.95^3^** |
|  | More (*n*=26) | **7** | **30.43^1^** |
|  | Total sample (*N*=122) | **24** | **25.26^2^** |
| Partner | Less (*n*=45) | **9** | **27.27^3^** |
|  | Same (*n*=51) | **9** | **23.07^2^** |
|  | More (*n*=26) | 2 | 8.70 |
|  | Total sample (*N*=122) | **20** | **21.05^3^** |
| Trainer | Less (*n*=45) | 6 | 18.18 |
|  | Same (*n*=51) | **7** | **17.95^3^** |
|  | More (*n*=26) | 2 | 8.70 |
|  | Total sample (*N*=122) | 15 | 15.79 |
| Sport Association | Less (*n*=45) | 3 | 9.09 |
|  | Same (*n*=51) | 3 | 7.69 |
|  | More (*n*=26) | **3** | **13.04^3^** |
|  | Total sample (*N*=122) | 9 | 9.47 |
| Team mates/Training partner | Less (*n*=45) | 3 | 9.09 |
|  | Same (*n*=51) | 2 | 5.13 |
|  | More (*n*=26) | **3** | **13.04^3^** |
|  | Total sample (*N*=122) | 8 | 8.42 |
| Sport Club | Less (*n*=45) | 1 | 3.03 |
|  | Same (*n*=51) | 1 | 2.56 |
|  | More (*n*=26) | 1 | 4.35 |
|  | Total sample (*N*=122) | 3 | 3.16 |
| No help | Less (*n*=45) | 0 | 0 |
|  | Same (*n*=51) | 2 | 5.13 |
|  | More (*n*=26) | 0 | 0 |
|  | Total sample (*N*=122) | 2 | 2.10 |
| Employer | Less (*n*=45) | 0 | 0 |
|  | Same (*n*=51) | 0 | 0 |
|  | More (*n*=26) | 1 | 4.35 |
|  | Total sample (*N*=122) | 1 | 1.05 |

*Note*. ^1^ = received help with the highest frequency for this subgroup; ^2^ = received help with the second highest frequency; ^3^ = received help with the third-highest frequency

Table 4

*Frequencies of wanted help for the complete sample and depending on motivational changes due to the lockdown*

| Wanted help | Change in motivation to exercise | *n* | *%* |
| --- | --- | --- | --- |
| Trainer | Less (*n*=35) | **5** | **15.15^3^** |
|  | Same (*n*=24) | **5** | **12.82^2^** |
|  | More (*n*=35) | **11** | **47.83^1^** |
|  | Total sample (*N*=94) | **21** | **22.11^1^** |
| Government/Country | Less (*n*=35) | **7** | **21.21^1^** |
|  | Same (*n*=24) | **4** | **10.26^3^** |
|  | More (*n*=35) | **9** | **39.13^2^** |
|  | Total sample (*N*=94) | **20** | **21.05^2^** |
| No wish for help | Less (*n*=35) | 4 | 12.12 |
|  | Same (*n*=24) | **6** | **15.38^1^** |
|  | More (*n*=35) | **5** | **21.74^3^** |
|  | Total sample (*N*=94) | **15** | **15.79^3^** |
| University | Less (*n*=35) | **7** | **21.21^1^** |
|  | Same (*n*=24) | 3 | 7.69 |
|  | More (*n*=35) | 3 | 13.04 |
|  | Total sample (*N*=94) | 13 | 13.68 |
| Family | Less (*n*=35) | 2 | 6.06 |
|  | Same (*n*=24) | 1 | 2.56 |
|  | More (*n*=35) | 2 | 8.70 |
|  | Total sample (*N*=94) | 5 | 5.26 |
| Sport team/Team mates | Less (*n*=35) | 1 | 3.03 |
|  | Same (*n*=24) | 3 | 7.69 |
|  | More (*n*=35) | 1 | 4.35 |
|  | Total sample (*N*=94) | 5 | 5.26 |
| Friends | Less (*n*=35) | 2 | 6.06 |
|  | Same (*n*=24) | 0 | 0 |
|  | More (*n*=35) | 2 | 8.70 |
|  | Total sample (*N*=94) | 4 | 4.21 |
| Sport Association | Less (*n*=35) | 2 | 6.06 |
|  | Same (*n*=24) | 1 | 2.56 |
|  | More (*n*=35) | 1 | 4.35 |
|  | Total sample (*N*=94) | 4 | 4.21 |
| Sport Club | Less (*n*=35) | 2 | 6.06 |
|  | Same (*n*=24) | 1 | 2.56 |
|  | More (*n*=35) | 0 | 0 |
|  | Total sample (*N*=94) | 3 | 3.16 |
| Sport Psychology | Less (*n*=35) | 2 | 6.06 |
|  | Same (*n*=24) | 0 | 0 |
|  | More (*n*=35) | 0 | 0 |
|  | Total sample (*N*=94) | 2 | 2.11 |
| Partner | Less (*n*=35) | 1 | 3.03 |
|  | Same (*n*=24) | 0 | 0 |
|  | More (*n*=35) | 1 | 4.35 |
|  | Total sample (*N*=94) | 2 | 2.11 |

*Note*. ^1^ = wanted help with the highest frequency for this subgroup; ^2^ = wanted help with the second highest frequency; ^3^ = wanted help with the third-highest frequency

Table 5

*Frequencies of joyful anticipation for the complete sample and depending on motivational changes due to the lockdown*

| Joyful anticipation about… | Change in motivation to exercise | *n* | *%* |
| --- | --- | --- | --- |
| Meeting social contacts | Less (*n*=97) | **29** | **87.88^1^** |
|  | Same (*n*=57) | 7 | 17.95 |
|  | More (*n*=65) | **22** | **95.65^1^** |
|  | Total sample (*N*=219) | **58** | **61.05^1^** |
| Leisure time activities | Less (*n*=97) | 11 | 33.33 |
|  | Same (*n*=57) | **14** | **35.90^1^** |
|  | More (*n*=65) | **15** | **65.22^2^** |
|  | Total sample (*N*=219) | **40** | **42.11^2^** |
| Sport with Others | Less (*n*=97) | 11 | 33.33 |
|  | Same (*n*=57) | **8** | **20.51^3^** |
|  | More (*n*=65) | 6 | 26.09 |
|  | Total sample (*N*=219) | **25** | **26.32^3^** |
| Normal Training conditions | Less (*n*=97) | **13** | **39.39^2^** |
|  | Same (*n*=57) | 3 | 7.69 |
|  | More (*n*=65) | **9** | **39.13^3^** |
|  | Total sample (*N*=219) | **25** | **26.32^3^** |
| Open sports facilities | Less (*n*=97) | **12** | **36.36^3^** |
|  | Same (*n*=57) | **9** | **23.08^2^** |
|  | More (*n*=65) | 3 | 13.04 |
|  | Total sample (*N*=219) | 24 | 25.26 |
| Normality | Less (*n*=97) | **12** | **36.36^3^** |
|  | Same (*n*=57) | 6 | 15.38 |
|  | More (*n*=65) | 3 | 13.04 |
|  | Total sample (*N*=219) | 21 | 22.11 |
| University/Education | Less (*n*=97) | 7 | 21.21 |
|  | Same (*n*=57) | 4 | 10.26 |
|  | More (*n*=65) | 3 | 13.04 |
|  | Total sample (*N*=219) | 14 | 14.74 |
| Competitions | Less (*n*=97) | 2 | 6.06 |
|  | Same (*n*=57) | 2 | 5.13 |
|  | More (*n*=65) | 2 | 8.70 |
|  | Total sample (*N*=219) | 6 | 6.32 |
| Travel | Less (*n*=97) | 0 | 0 |
|  | Same (*n*=57) | 2 | 5.13 |
|  | More (*n*=65) | 2 | 8.70 |
|  | Total sample (*N*=219) | 4 | 4.21 |
| Training camps | Less (*n*=97) | 0 | 0 |
|  | Same (*n*=57) | 2 | 5.13 |
|  | More (*n*=65) | 0 | 0 |
|  | Total sample (*N*=219) | 2 | 2.11 |

*Note*. ^1^ = joyful anticipation with the highest frequency for this subgroup; ^2^ = joyful anticipation with the second highest frequency; ^3^ = joyful anticipation with the third-highest frequency
